# Novel piplartine-containing ruthenium complexes: synthesis, cell growth inhibition, apoptosis induction and ROS production on HCT116 cells

**DOI:** 10.18632/oncotarget.22248

**Published:** 2017-11-01

**Authors:** Cinara O. D’Sousa Costa, João H. Araujo Neto, Ingrid R.S. Baliza, Rosane B. Dias, Ludmila de F. Valverde, Manuela T.A. Vidal, Caroline B.S. Sales, Clarissa A.G. Rocha, Diogo R.M. Moreira, Milena B.P. Soares, Alzir A. Batista, Daniel P. Bezerra

**Affiliations:** ^1^ Gonçalo Moniz Institute, Oswaldo Cruz Foundation (IGM-FIOCRUZ/BA), Salvador, Bahia, 40296-710, Brazil; ^2^ Department of Chemistry, Federal University of São Carlos, São Carlos, São Paulo, 13561-901, Brazil; ^3^ Department of Biomorphology, Institute of Health Sciences, Federal University of Bahia, Salvador, Bahia, 40110-902, Brazil; ^4^ Center of Biotechnology and Cell Therapy, Hospital São Rafael, Salvador, Bahia, 41253-190, Brazil

**Keywords:** piplartine, piperlongumine, ruthenium complexes, ROS, apoptosis

## Abstract

Piplartine (piperlongumine) is a plant-derived molecule that has been receiving intense interest due to its anticancer characteristics that target the oxidative stress. In the present paper, two novel piplartine-containing ruthenium complexes [Ru(piplartine)(dppf)(bipy)](PF_6_)_2_ (1) and [Ru(piplartine)(dppb)(bipy)](PF_6_)_2_ (2) were synthesized and investigated for their cellular and molecular responses on cancer cell lines. We found that both complexes are more potent than metal-free piplartine in a panel of cancer cell lines on monolayer cultures, as well in 3D model of cancer multicellular spheroids formed from human colon carcinoma HCT116 cells. Mechanistic studies uncovered that the complexes reduced the cell growth and caused phosphatidylserine externalization, internucleosomal DNA fragmentation, caspase-3 activation and loss of the mitochondrial transmembrane potential on HCT116 cells. Moreover, the pre-treatment with Z-VAD(OMe)-FMK, a pan-caspase inhibitor, reduced the complexes-induced apoptosis, indicating cell death by apoptosis through caspase-dependent and mitochondrial intrinsic pathways. Treatment with the complexes also caused a marked increase in the production of reactive oxygen species (ROS), including hydrogen peroxide, superoxide anion and nitric oxide, and decreased reduced glutathione levels. Application of N-acetyl-cysteine, an antioxidant, reduced the ROS levels and apoptosis induced by the complexes, indicating activation of ROS-mediated apoptosis pathway. RNA transcripts of several genes, including gene related to the cell cycle, apoptosis and oxidative stress, were regulated under treatment. However, the complexes failed to induce DNA intercalation. In conclusion, the complexes are more potent than piplartine against different cancer cell lines and are able to induce caspase-dependent and mitochondrial intrinsic apoptosis on HCT116 cells by ROS-mediated pathway.

## INTRODUCTION

Colon and rectal carcinoma is a disease with high frequency and lethality. In 2012, 1.4 million new cases were diagnosed and almost 694,000 deaths estimated worldwide [1]. Current chemotherapeutic drugs are an important form of treatment, but they have clinically serious toxicities and the cancer cells still can acquire resistance to them; therefore, the development of novel cytotoxic agents remains a great challenge [2].

Piplartine (piperlongumine) is an alkaloid, which is found in some *Piper* species. We and along with other research groups have been investigating the anticancer potential of piplartine, and some antineoplastic characteristics have been assigned to this molecule, including potent cytotoxic, genotoxic, antitumor, antiangiogenic and antimetastatic properties, as well as attractive good bioavailability and safety [3–18]. Historically, much attention has been given to this molecule after its cytotoxicity and ability to induce the production of reactive oxygen species (ROS) selectively in cancer cells were published by Raj et al. [12]. Later, the anticancer potential of piplartine and its analogs, alone or in combination, including combination with paclitaxel, cisplatin, gemcitabine and curcumin, have been extensively explored [10, 16, 19–21]. These studies reported the ability of piplartine to induce apoptosis and/or autophagy through modulation of the PI3K/Akt/mTOR, NF-κB, JAK1,2/STAT3 and/or JNK pathways in cancer cells [14, 15, 22–24]. In addition, piplartine is a direct TrxR1 inhibitor and can inhibit cell migration/invasion via ROS/ER/MAPKs/CHOP axis [25, 26].

Several ruthenium complexes exhibit potent cytotoxic activity to cancer cells [27–29]. Moreover, selected ruthenium complexes are under phase I or II clinical trials, with promising results [30, 31]. Interestingly, the structure of the ligands bound to the metal is important for the activity of these complexes. Consequently, several organic molecules have been used as ligands to form complexes with ruthenium, aiming at improving their cytotoxic activity. Therefore, we have investigated for the first time the cellular and molecular responses of two novel piplartine-containing ruthenium complexes [Ru(piplartine)(dppf)(bipy)](PF_6_)_2_ (**1**) and [Ru(piplartine)(dppb)(bipy)](PF_6_)_2_ (**2**) (dppf = 1,1-bis(diphenylphosphino) ferrocene; dppb = 1,4-bis(diphenylphosphino)butane and bipy = 2,2’-bipyridine), on human colon carcinoma HCT116 cells.

## RESULTS

### Synthesis of novel piplartine-containing ruthenium complexes

**Figure 1:**
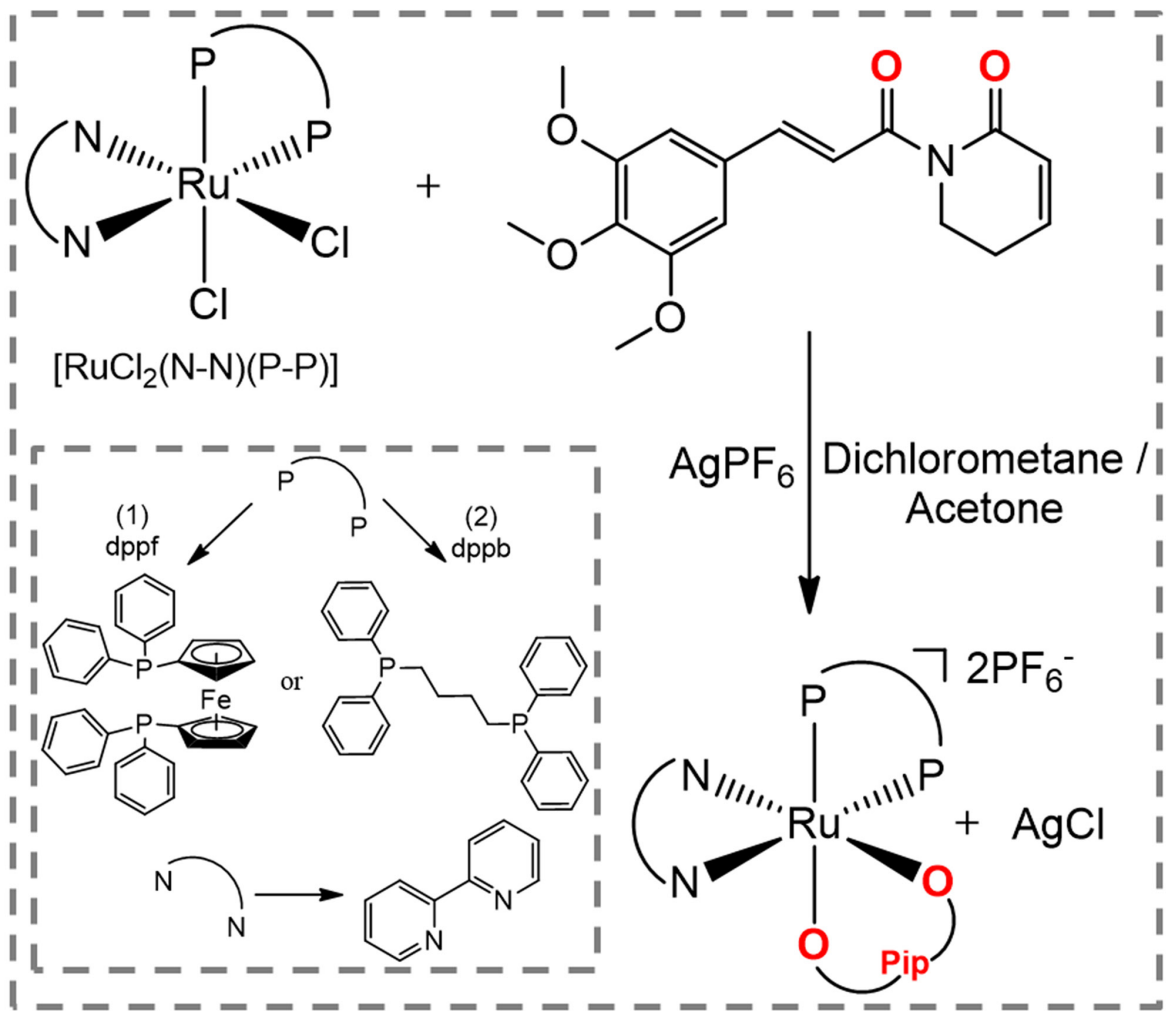
Route for the synthesis of complexes [Ru(piplartine)(dppf)(bipy)](PF_6_)_2_ (1) and [Ru(piplartine)(dppb)(bipy)](PF_6_)_2_ (2)

The novel piplartine-containing ruthenium complexes were obtained using two different precursors of type [RuCl_2_(N-N)(P-P)] (N-N = 2,2’-bipyridine (bipy); P-P = 1,1’-bis(diphenylphosphino) ferrocene (dppf) for complex **1** (heterometallic), and 1,4-bis (diphenylphosphino)butane (dppb) for complex **2** (monometallic), as indicated in Figure 1. Silver hexafluorophosphate was employed in order to sequester the precursor’s chlorido, allowing the coordination of the piplartine ligand to the metal center, and forming the insoluble AgCl salt, which was easily removed by filtration. The employment of dry non-coordinating solvent (acetone or dichloromethane), during the syntheses of the complexes is necessary to avoid the formation of by products of the reaction. Both complexes were prepared in good yields, 87% for complex **1** and 88% for complex **2**, as orange solids, stable under light and air.

The piplartine molecule is an uncharged ligand and its coordination to the Ru(II) precursors results in dicationic complexes, which precipitate in the salt form with two hexafluorophosphate anion (PF_6_^-^) acting as counterions. The formation of dicationic complexes was confirmed by molar conductivity measurements, performed in acetone, displaying typical solution of 2:1 electrolyte, with conductivity range [32] between 160–200 S cm^2^ mol^-1^. The elemental analyses of the complexes are consistent with their proposed formulas.

The infrared spectra of the complexes show the typical ν(C=O) carboxyl stretching frequencies, at 1650 cm^-1^ for complex **1**, 1651 cm^-1^ for complex **2**, while the metal-free piplartine ligand displays this stretching mode at 1686 cm^-1^. The difference between the metal-free and coordinated ν(C=O) piplartine values (Δν = 36 cm^-1^) is indicative of a bidentate binding mode of the ligand, through the carbonyl groups [33]. Strong bands are present in the spectra of the piplartine and of the precursor complexes in the region of 1600–1300 cm^-1^, characteristics of νC=N and νC=C stretching vibrations. The complexes **1** and **2** exhibit νRu-P stretching bands in the range of 520–508 cm^-1^. Also, the νRu-N and νRu-O stretching vibrations occur as weak bands in the region of low energy, around 450–350 cm^-1^. For both complexes, the characteristic P–F stretch of the PF_6_^-^ counterion are at 843 cm^-1^. Most of the vibrational modes observed in the infrared spectra of the complexes are characteristic of the diphosphine/bipyridine ligands, occurring practically at the same frequencies observed for the precursors of type *cis*-[RuCl_2_(P-P)(N-N)] [34–37].

**Figure 2:**
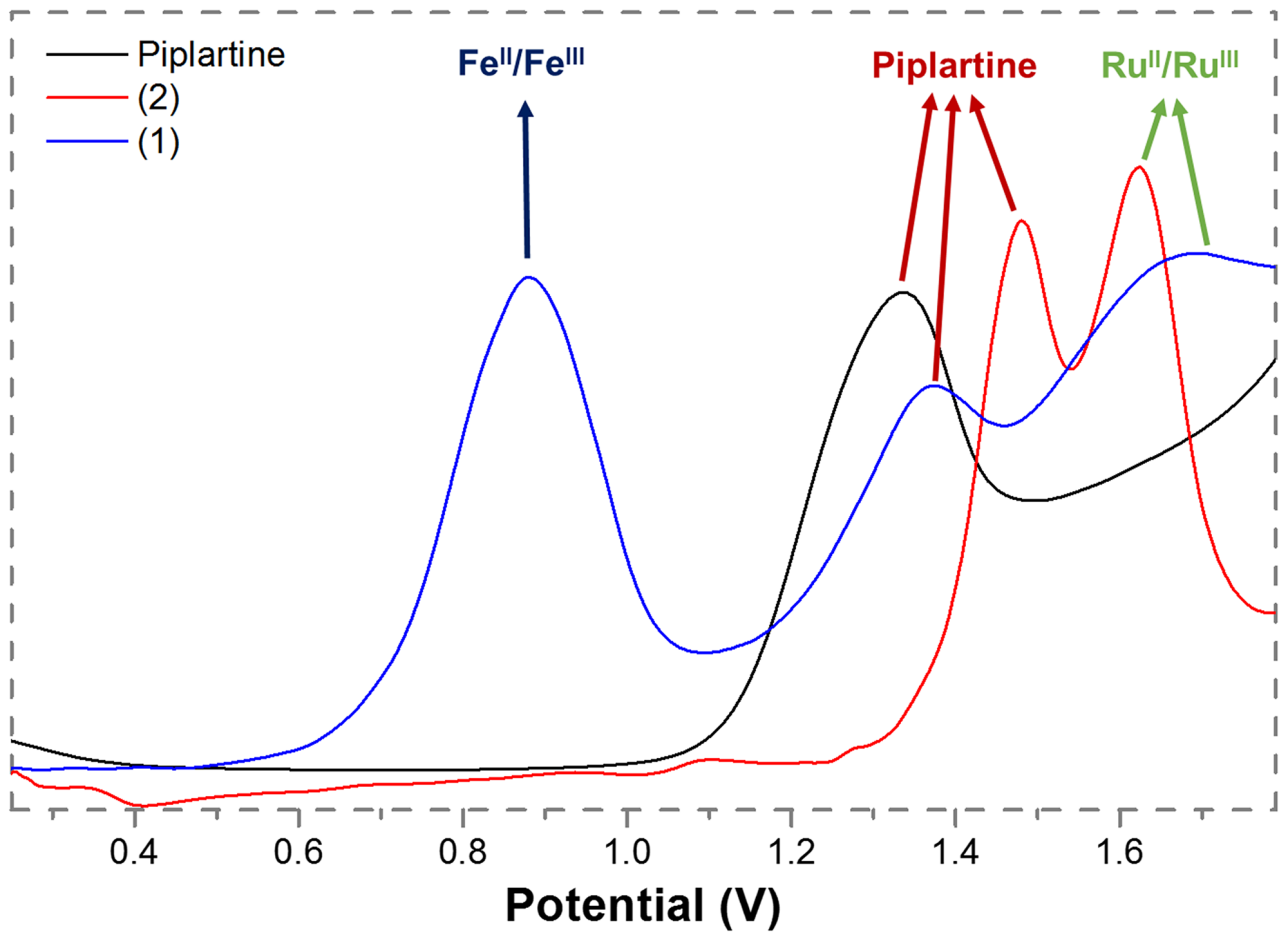
Diferential pulse voltamogramms of complexes 1 and 2 (dichlorometane solution, working and auxiliary electrodes were stationary Pt, and the reference electrode was Ag/AgCl, 0.10 M Bu_4_NClO_4_)

The electrochemical behavior of the complexes **1** and **2** are similar to those found for other Ru(II)/diphosphine/diimine complexes [34–36]. These experiments were performed by cyclic (CV) and differential pulse voltammetry (DPV) techniques, in dichloromethane solutions. The DPV oxidation of the complexes and of the metal-free piplartine are represented in Figure 2. The complex **1**, which is a bimetallic compound, exhibits three oxidation processes: the first one, which is quasi-reversible ((I_pa_/I_pc_ = 1.2, E_pa_ = 0.88 V) belongs to the Fe(II)/Fe(III) oxidation, while the second one, irreversible, with E_pa_ = 1.37 V, belongs to the oxidation process of the piplartine. This process is also observed for the metal-free ligand (Figure 2). The third process, a irreversible process, is assigned to one-electron Ru(II)/Ru(III), with E_pa_ 1.68 V (Table 1). The complex **2** exhibits two processes, the first one, irreversible, at 1.48 V (E_pa_), belongs to the oxidation of the piplartine ligand and the second one, quasi-reversible, at 1.62 V (E_pa_), belongs to the Ru(II)/Ru(III). The process assigned to piplartine refers to the oxidation of the trimethoxybenzene group, where the proposed mechanism involves the formation of free radicals [38]. The E_pa_ value of Ru(II)/Ru(III) oxidation processes for the precursor *cis*-[RuCl_2_(dppb)(bipy)] is observed around 0.65 V [34, 39], clearly indicating that the substitution of the two chloridos by an uncharged chelate ligand (piplartine), stabilizing the Ru(II) center by approximately 1.0 V. This stabilization is plausible, due to the replacement of two σ and π donor chlorido ligands by an uncharged chelating piplartine, which increases the oxidation potential of the metal center.

**Table 1:** Attributions of vibrational frequencies (cm) corresponding to the carboxyl group in the metal-free and coordinated piplartine, ^31^P(^1^H) shift NMR (d_6_-acetone) and electrochemical data

	νC=O (cm)	Δν (cm)	δ ^31^P(^1^H) (ppm)	^2^*J*_*P-P*_ (Hz)	E_pa_/E_pc_ (V) Fe(II)/Fe(III)	E_pa_/E_pc_ (V) Ru(II)/Ru(III)	E_pa_/E_pc_ (V) piplartine
Piplartine	1686	-	-	-	-	-	1.33 / -
**1**	1650	36	39.1; 39.3 43.1; 45.9	29.6 30.3	0.88 / 0.75	1.62 / -	1.37 / -
**2**	1651	35	39.4; 42.3 41.0; 41.7	32.6 34.9	-	1.62 / 1.50	1.48 / -

**Figure 3:**
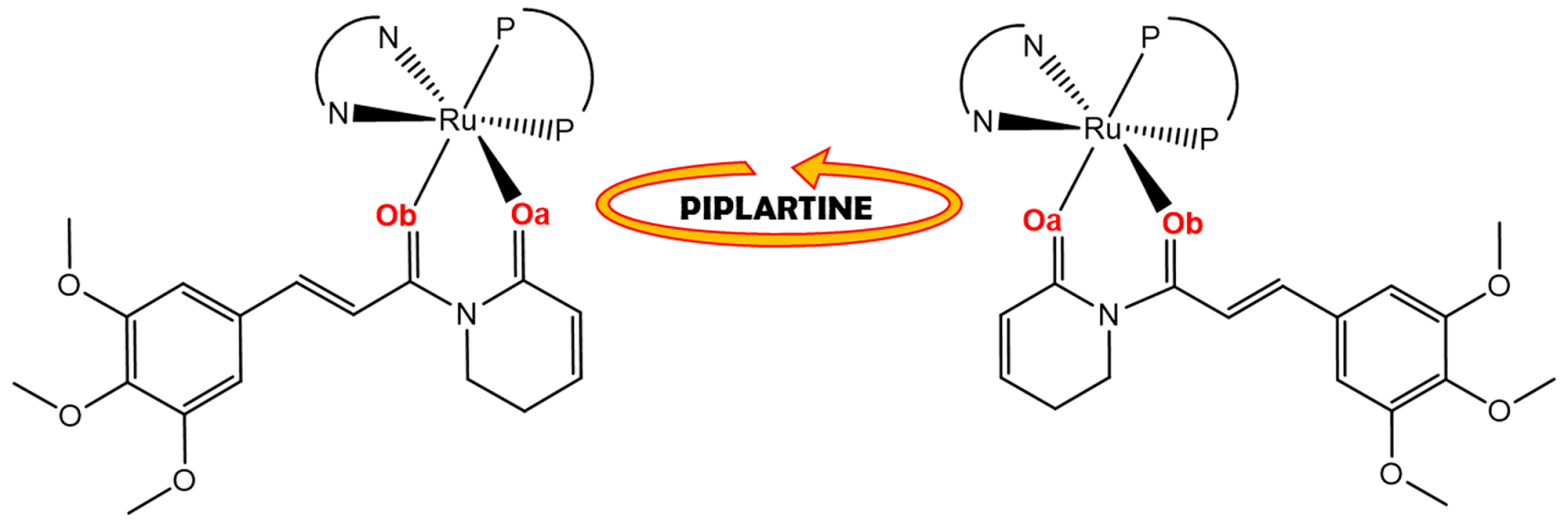
Representation of isomers resulting from bond rotation of piplartine around the metal center

**Figure 4:**
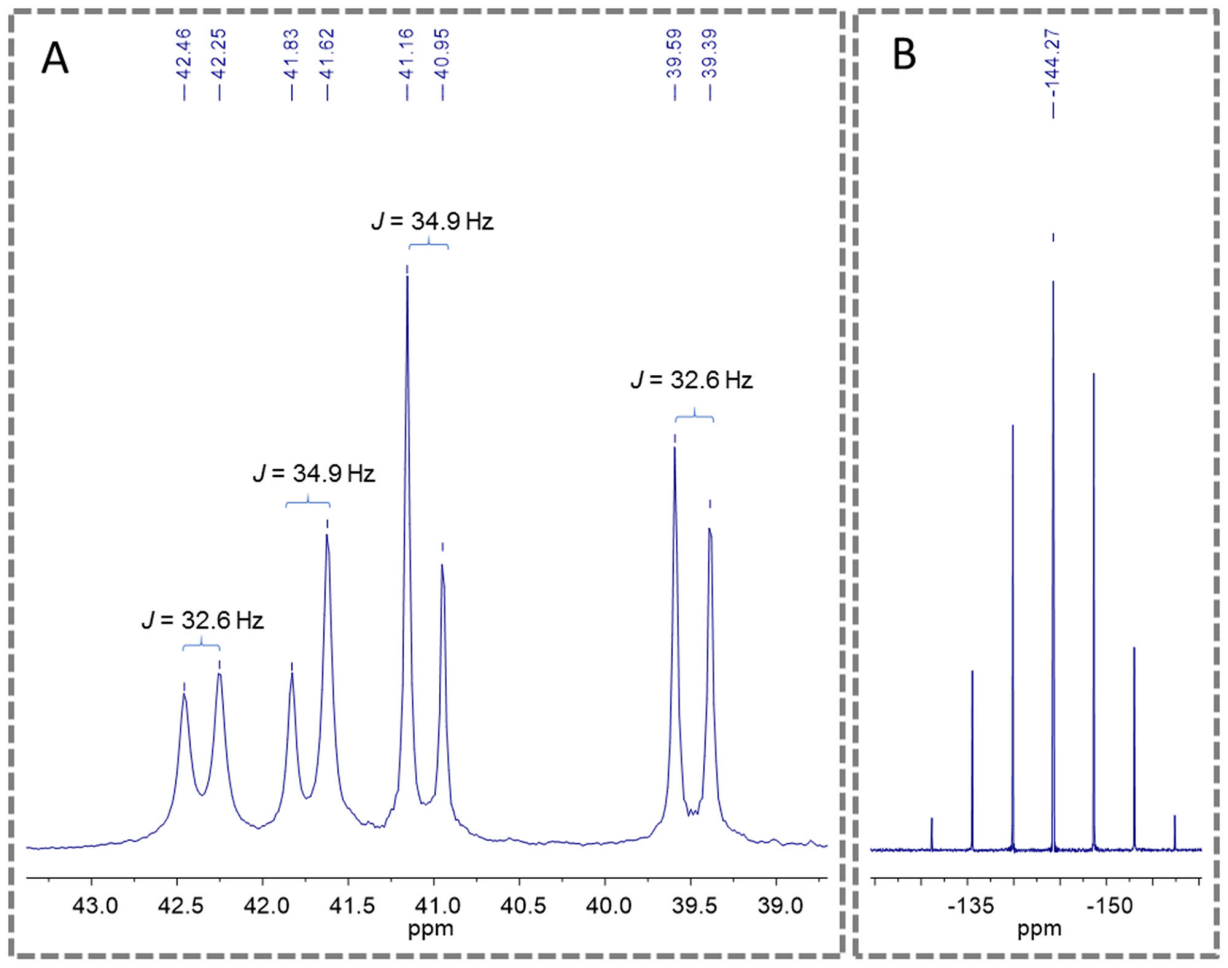
^31^P{^1^H} NMR spectra of the complex 2 (d_6_-acetone), where (A) show the doublets of the phosphorous of diphosphine and (B) show the multiplet signal of PF_6_^-^

An important aspect observed to the novel piplartine-containing ruthenium complexes is the presence of isomers generated from the position of the piplartine ligand, as shown in Figure 3. For one isomer, the coordinated oxygen – O_a_ is *trans* to the nitrogen from the bipyridine ligand, while the O_b_ is *trans* to the phosphorous atom from the diphosphine. The second isomer has the opposite, the O_a_ *trans* to the phosphorous atom from diphophine and the O_b_, *trans* to the nitrogen from the bipyridine. The presence of the two isomers was observed by using the multiatomic NMR technique, by the duplication of signals in the spectra. Thus, in the ^31^P{^1^H} NMR spectra of the complexes **1** and **2**, two pairs of doublets were observed, where each pair is consistent with an AB pattern with specific *J*-coupling (Table 1). This system indicates the presence of two inequivalent phosphorus atoms for each isomer. The signals, in the region of 39 to 45 ppm for complex **1** and 39 to 41 ppm for complex **2** (Figure 4), are consistent with a geometry where one of the nitrogens of the 2,2’-bipyridine is *trans* to one phosphorus atom of dppb/dppf, for both complexes [40], and the second one is *trans* to oxygen of carboxyl groups, O_a_ or O_b_ (isomers), as shown in Figure 3. For both complexes the integral for the phosphorous signals is the same, showing that isomers ratio at the final product is 1:1.

**Figure 5:**
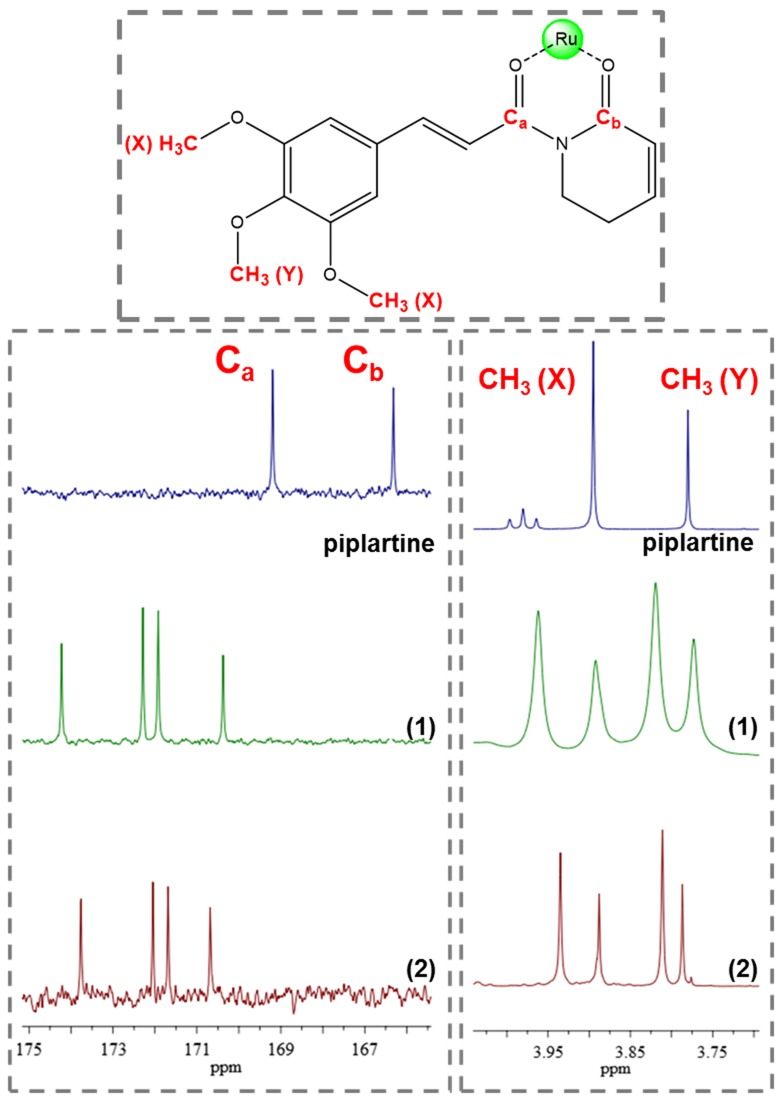
^13^C and ^1^H NMR spectrum of complexes 1 and 2 with amplification in the region of methoxyl group displaying the signal duplication, indicative of isomers (d_6_-acetone)

The ^1^H NMR spectra of the complexes, in d_6_-acetone, display signals correspondent to the hydrogen atoms of the ligands diphosphine, bipyridine and piplartine, exhibiting several duplicate signals, confirming the presence of the isomers in solution [41]. The metal-free ligand, piplartine, displays two broad singlets, at 3.78 and 3.90 ppm, referent to the methoxy groups present in the molecule. For the complexes **1** and **2**, these signals present similar chemical shift, however both signal are duplicate, as shown in Figure 5. The aromatic hydrogens of the phenyl group of the dppf (**1**) and of the dppb (**2**) ligands are in the typical range 6.0–7.9 ppm. The complex **1** exhibits the expected shielded aromatic signals corresponding to the hydrogens of the ferrocene rings of the dppf ligand at 4.3 – 5.3 ppm. In addition, both complexes exhibited the expected duplicated deshielded doublets corresponding to the ortho hydrogens of the 2,2’-bipiridinic ligand at 8.45 – 9.05 ppm for complex **1** and 8.55 – 8.85 ppm for complex **2**.

The ^13^C NMR spectra of the complexes display four signals around 170 – 175 ppm, typical of the coordinated C=O group. This signal is deshielded, compared with the observed for the metal-free piplartine ligand in where they occur at 165 and 168 ppm, indicating that oxygen of carboxyl group is coordinated to the metal. In agreement with the previously NMR techniques (^31^P{^1^H} and ^1^H NMR) employed, all signals are also duplicate, due the presence of isomers, as shown to the coordinated carbonyl ^13^C NMR signals in Figure 5.

The ^31^P{^1^H} NMR technique is also a versatile tool to study the stability of the complexes **1** and **2** in different solvents. The complexes display instability when dissolved in coordinating solvents (DMSO, methanol or DMF). Thus, when the complexes are dissolved in these solvents the ligand piplartine is quickly and completely labilized from the metal center. Therefore, in noncoordinating solvents (acetone, dichloromethane or chloroform) the complexes present stability, for at least 72 h. In an acetone/water (1:9) mixture, labilization of piplartine occurs gradually (red arrow), and after 24 h the complete labilization of piplartine is observed, showing the presence of only one species in solution [probably the [Ru(H_2_O)_2_(dppb)(bipy)]^2+^ species (orange arrow) (Supplementary Figure 1)], which displays only a pair of doublets, at about 37 and 48 ppm.

The HPLC chromatogram of complex **2** shows the instability of this compound in the eluent used, displaying different peaks, which can be attributed to the isomers of complex **2** or the solvolysis/hydrolysis products such as the metal-free piplartine, [Ru(dppb)(bipy)(MeOH)_2_]^2+^ and [Ru(dppb)(bipy)(H_2_O)_2_]^2+^ or [Ru(dppb)(bipy)(MeOH)(H_2_O)]^2+^ species and other byproducts (Supplementary Figure 2). The HPLC experiments were performed using methanol/water solution (57/43) as mobile phase at isocratic model and the column ODS-C18 (5 mm; 250 x 4.6 mm; Shimadzu).

### Piplartine-containing ruthenium complexes display potent cytotoxicity against a panel of cancer cell lines

The cytotoxicity of both piplartine-containing ruthenium complexes was screened against a panel of different histological types of cancer cell lines (HCT116, HepG2, HSC-3, SCC-4, SCC-9, HL-60, K-562 and B16-F10) and against two non-cancer cells (MRC-5 and PBMC) after 72h of incubation using the alamar blue assay. Table 2 shows the results obtained. Both complexes presented cytotoxic activity more potent than metal-free piplartine. Complex **1** presented IC_50_ values ranging from 0.6 to 4.4 μM for cancer cell lines HSC3 and SCC9, respectively. Complex **2** presented IC_50_ values ranging from 1.3 to 6.8 μM for cancer cell lines HSC3 and SCC9, respectively. On the other hand, piplartine presented IC_50_ values ranging from 6.3 to 18.6 μM for cancer cell lines HepG2 and K562, respectively. Complex **1** was more potent than metal-free piplartine on HCT116 (4-fold), HepG2 (4-fold), HSC3 (12-fold), SCC4 (5-fold), SCC9 (4-fold), HL-60 (4-fold), K562 (5-fold) and B16-F10 (4-fold). Complex **2** was more potent that metal-free piplartine on HepG2 (3-fold), HSC3 (6-fold), SCC4 (3-fold), SCC9 (2-fold), HL-60 (3-fold), K562 (3-fold) and B16-F10 (3-fold). Doxorubicin presented IC_50_ values ranging from 0.02 to 2.6 μM for cancer cell lines B16-F10 and SCC9, respectively. Oxaliplatin presented IC_50_ values ranging from 0.6 to 7.7 μM for cancer cell lines HL-60 and SCC4, respectively. The precursors of type [RuCl_2_(N-N)(P-P)] (N-N = diimines; P-P = diphosphines) had been previously tested and exhibited only weak cytotoxicity (IC_50_ > 15 μM) [42, 43], and was not tested in the present paper.

**Table 2:** Cytotoxic activity of piplartine-containing ruthenium complexes

Cells	IC_50_ in μM				
	DOX	OXA	PL	1	2
Cancer cells					
HCT116	0.2 0.1 - 0.3	4.1 2.3 - 5.5	6.4 3.2 – 9.5	1.7 1.5 - 2.0	5.5 4.7 - 6.4
HepG2	0.2 0.2 - 0.3	2.2 1.3 – 3.8	6.3 4.4 – 8.8	1.7 1.4 - 2.2	1.9 1.4 - 3.5
HSC-3	0.5 0.3 - 0.6	3.3 1.4 – 7.8	7.4 3.1 – 11.3	0.6 0.5 - 0.9	1.3 0.8 - 2.3
SCC-4	2.1 1.7 - 2.6	7.7 4.6 – 13.0	15.5 11.3 - 19.8	3.2 2.2 - 4.8	5.4 3.9 - 7.3
SCC-9	2.6 2.0 - 3.3	N.d.	16.5 14.2 - 19.0	4.4 3.3 - 5.9	6.8 5.3 - 8.8
HL-60	0.2 0.2 - 0.3	0.6 0.1 – 0.8	13.3 4.1 – 18.7	3.4 1.8 - 6.3	4.5 2.9 – 7.0
K-562	1.0 0.6 - 1.8	1.0 0.1 – 1.3	18.6 11.6 – 23.9	3.5 2.9 - 4.4	5.8 4.9 - 6.7
B16-F10	0.02 0.01 - 0.07	2.2 1.2 - 4.1	10.6 6.9 - 16.1	2.8 2.0 - 3.8	4.1 3.0 - 5.4
Non-cancer cells					
MRC-5	1.3 1.0 - 1.5	1.3 1.0 - 2.2	17.3 11.3 - 25.5	3.4 2.8 – 4.0	6.3 4.3 - 9.2
PBMC	5.4 3.1 - 9.4	9.4 6.5 - 11.4	34.2 28.1 - 43.9	1.8 1.1 - 3.2	3.2 1.8 - 5.9

Data are presented as IC_50_ values in μM and their respective 95% confidence interval obtained by nonlinear regression from at the least three independent experiments performed in duplicate, measured by alamar blue assay after 72h of incubation. Cancer cells: HCT116 (human colon carcinoma); HepG2 (human hepatocellular carcinoma); HSC-3 (human oral squamous cell carcinoma); SCC-4 (human oral squamous cell carcinoma); SCC-9 (human oral squamous cell carcinoma); HL-60 (human promyelocytic leukemia); K-562 (human chronic myelogenous leukemia); and B16-F10 (murine melanoma). Non-cancer cells: MRC-5 (human lung fibroblast) and PBMC (human peripheral blood mononuclear cells). Doxorubicin (DOX), oxaliplatin (OXA) and piplartine (PL) were used as the positive controls. N.d. Not determined.

The IC_50_ value for non-cancer cells was 3.4 and 1.8 μM for the complex **1**, 6.3 and 3.2 μM for the complex **2** and 17.3 and 34.2 μM for piplartine for MRC-5 and PBMC cells, respectively. In addition, the IC_50_ value for non-cancer cells was 1.3 and 5.4 μM for doxorubicin and 1.3 and 9.4 μM for oxaliplatin for MRC-5 and PBMC cells, respectively. The Supplementary Table 1 shows the selectivity index (SI) calculated. The SI was calculated using the following formula: SI = IC_50_ [non-cancer cells]/IC_50_ [cancer cells]. The SI is a well-characterized method to estimate the therapeutic range of a drug and to identify drug candidates for further studies. Although the complexes were more cytotoxic to non-cancer cells than piplartine, they exhibit selectivity index similar to piplartine and the positive controls doxorubicin and oxaliplatin, which are clinically useful drugs in the treatment of cancer.

In a new set of experiment, human colon carcinoma HCT116 cell line was used as a cellular model, since it was among the most sensitive cell lines to the complexes tested.

**Figure 6:**
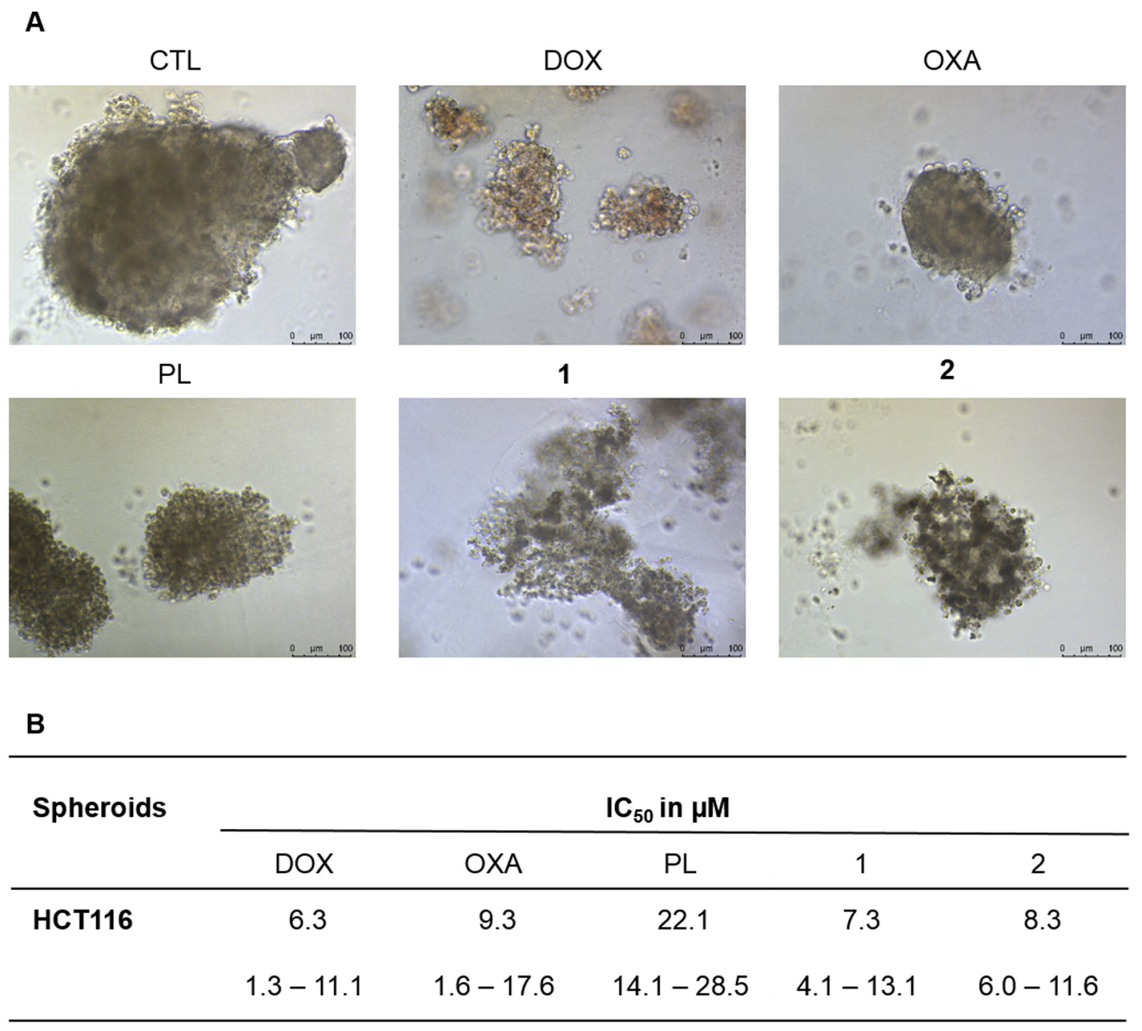
Effect of piplartine-containing ruthenium complexes in 3D *in vitro* model of cancer multicellular spheroids formed from HCT116 cells **(A)** Cells examined by light microscopy (bar = 100 μm) at the highest concentration tested. **(B)** IC_50_ values in μM and their respective 95% confidence interval obtained by nonlinear regression from at the least three independent experiments performed in duplicate, measured by alamar blue assay after 72h of incubation. The negative control (CTL) was treated with the vehicle (0.1% of a solution containing 70% sorbitol, 25% tween 80 and 5% water) used for diluting the compounds tested. Doxorubicin, oxaliplatin and piplartine were used as the positive controls.

In complementary to 2D cell monolayer cultures, we also assessed the cytotoxicity of piplartine-containing ruthenium complexes in an *in vitro* three-dimensional (3D) model of cancer multicellular spheroids formed from HCT116 cells. The cells in the spheroids showed morphological changes, indicating an effective drug permeability and cytotoxicity in the 3D culture (Figure 6A). After 72 h treatment, the IC_50_ of the complexes **1** and **2** were estimated to be 7.3 and 8.3 μM, respectively (Figure 6B), while piplartine presented IC_50_ of 22.1 μM. Based on this, the complexes **1** and **2** were approximately 3 and 2 fold more potent than metal-free piplartine, respectively. Doxorubicin and oxaliplatin showed IC_50_ of 6.3 and 9.3 μM, respectively.

### Piplartine-containing ruthenium complexes induce caspase-dependent and mitochondrial intrinsic apoptosis on HCT116 cells

**Figure 7:**
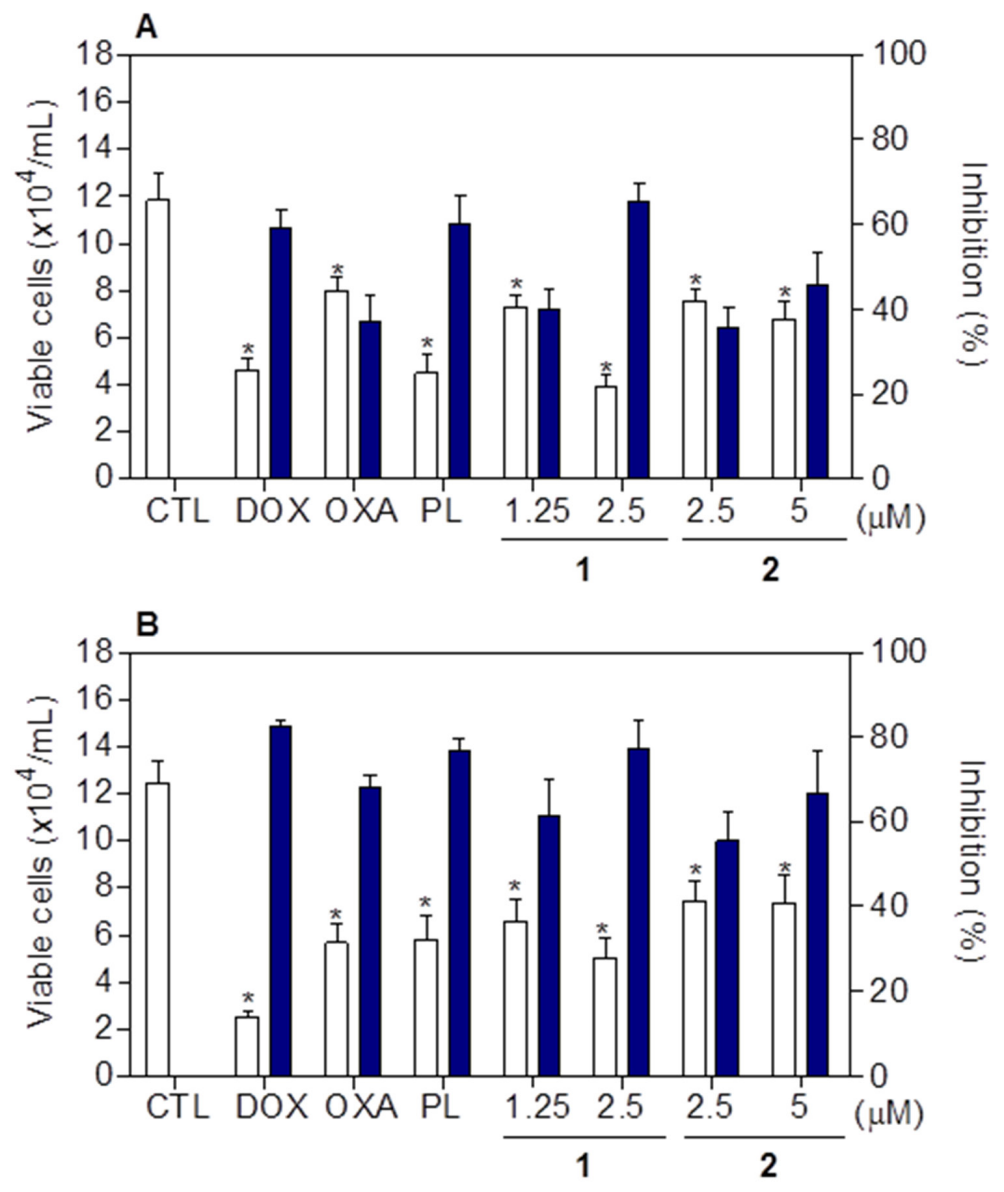
Effect of piplartine-containing ruthenium complexes in the cell viability of HCT116 cells determined by trypan blue staining after 24 **(A)** and 48 **(B)** h of incubation. The white bars represent number of viable cells (x104cells/mL) and the blue bars represent cell inhibition (%). The negative control (CTL) was treated with the vehicle (0.1% of a solution containing 70% sorbitol, 25% tween 80 and 5% water) used for diluting the compounds tested. Doxorubicin (DOX, 1 μM), oxaliplatin (OXA, 3 μM) and piplartine (PL, 10 μM) were used as the positive controls. Data are presented as the mean ± S.E.M. of three independent experiments performed in duplicate. * *p* < 0.05 compared with the negative control by ANOVA followed by Student Newman-Keuls test.

Cell viability after treatment with complexes **1** and **2** was determined by trypan blue exclusion assay on HCT116 cells, after 24 and 48h of incubation. Both complexes significantly reduced (*p* < 0.05) the number of viable cells (Figure 7). At concentrations of 1.25 and 2.5 μM, complex **1** reduced the number of viable cells by 40.2 and 65.4% after 24 h, and 61.5 and 77.1% after 48 h, respectively. Complex **2** reduced the number of viable cells by 35.5 and 45.8% at 2.5 and 5 μM after 24h, and 55.5 and 66.9% after 48h, respectively. None of complexes induced significant (*p* > 0.05) increase in the non-viable cells. Doxorubicin, oxaliplatin and piplartine also reduced the number of viable cells after 24 and 48h of incubation.

Regarding cell morphology, HCT116 cells treated with both complexes at all concentrations induced reduction in the cell volume, chromatin condensation and fragmentation of the nuclei. This effect became more pronounced at the higher concentrations and at the longer time of incubation (Supplementary Figure 3A). Doxorubicin, oxaliplatin and piplartine also induced cell shrinkage, chromatin condensation and nuclear fragmentation. Furthermore, both complexes caused cell shrinkage, as observed by the decrease in forward light scatter (FSC), as well as nuclear condensation, indicated by a transient increase in side scatter (SCC) (Supplementary Figures 3B, 4A and 4B). These morphological alterations indicate that the complexes were inducing apoptosis.

DNA content was examined by flow cytometry on HCT116 cells (Table 3). All DNA that was sub-diploid in size (sub-G_0_/G_1_) was considered fragmented. Both complexes at higher concentrations caused a significant DNA fragmentation (*p* < 0.05). Complex **1** led to 31.8 and 45.8% DNA fragmentation at the higher concentration after 24 and 48h, respectively. Complex **2** induced 23.6 and 31.9% DNA fragmentation at the higher concentration after 24 and 48 h, respectively. Piplartine induced 20.3 and 33.9% DNA fragmentation after 24 and 48 h, respectively. Doxorubicin caused cell cycle arrest at the phase G_2_/M that was followed by DNA fragmentation. Oxaliplatine also induced DNA fragmentation.

**Table 3:** Effect of piplartine-containing ruthenium complexes in the DNA content of HCT116 cells

Treatment	Concentration (μM)	DNA content (%)			
		Sub-G_0_/G_1_	G_0_/G_1_	S	G_2_/M
After 24h of incubation					
CTL	-	5.0 ± 0.9	46.6 ± 1.8	18.7 ± 1.6	28.0 ± 1.6
DOX	1	5.2 ± 1.4	22.1 ± 2.4	9.8 ± 0.8^*^	59.0 ± 2.8^*^
OXA	3	14.3 ± 3.6	38.4 ± 5.4	16.9 ± 2.0	25.4 ± 2.1
PL	10	20.3 ± 1.8^*^	22.7 ± 0.6	21.4 ± 2.3	21.5 ± 2.9
**1**	1.25	9.1 ± 2.4	42.5 ± 1.7	21.9 ± 2.5	22.4 ± 1.1
	2.5	31.8 ± 1.4^*^	28.6 ± 5.9	11.5 ± 2.5	12.7 ± 1.7^*^
**2**	2.5	8.8 ± 1.4	45.1 ± 1.3	18.7 ± 1.8	22.9 ± 1.9
	5	23.6 ± 7.9^*^	41.0 ± 4.6	15.6 ± 1.8	19.0 ± 2.9
After 48h of incubation					
CTL	-	3.9 ± 0.8^*^	47.5 ± 3.3	20.1 ± 2.6	22.9 ± 1.1
DOX	1	21.8 ± 5.4^*^	15.8 ± 2.0^*^	10.4 ± 1.1^*^	49.0 ± 5.9^*^
OXA	3	14.8 ± 1.3^*^	44.2 ± 2.2	17.4 ± 1.9	21.2 ± 1.0
PL	10	33.9 ± 5.8^*^	27.0 ± 2.9^*^	16.4 ± 2.2	21.9 ± 0.9
**1**	1.25	13.5 ± 1.0	39.4 ± 1.8	20.1 ± 1.4	21.5 ± 0.8
	2.5	45.8 ± 8.8^*^	30.0 ± 5.8^*^	13.5 ± 1.5	9.0 ± 3.2^*^
**2**	2.5	15.7 ± 3.1	33.7 ± 4.2^*^	21.2 ± 2.6	20.5 ± 2.5
	5	31.9 ± 4.2^*^	18.0 ± 2.7^*^	18.0 ± 1.3	15.7 ± 2.6

Data are presented as the mean ± S.E.M. of three independent experiments performed in duplicate. The negative control (CTL) was treated with the vehicle (0.1% of a solution containing 70% sorbitol, 25% tween 80 and 5% water) used for diluting the compounds tested. Doxorubicin (DOX), oxaliplatin (OXA) and piplartine (PL) were used as the positive controls. Ten thousand events were evaluated per experiment and cellular debris was omitted from the analysis. ^*^ *p* < 0.05 compared with the negative control by ANOVA followed by Student Newman-Keuls Test.

**Figure 8:**
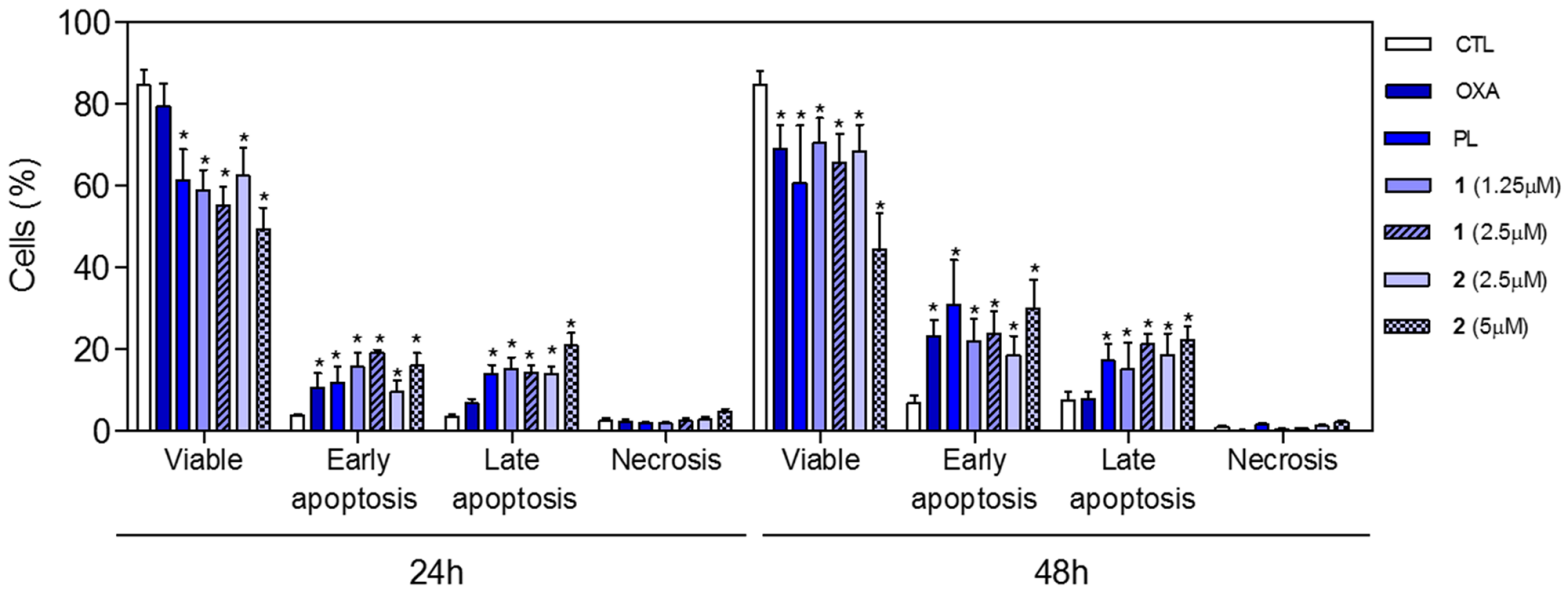
Effect of piplartine-containing ruthenium complexes in the induction of apoptosis on HCT116 cells determined by flow cytometry using annexin V-FITC/PI staining after 24 and 48h of incubation The negative control (CTL) was treated with the vehicle (0.1% of a solution containing 70% sorbitol, 25% tween 80 and 5% water) used for diluting the compounds tested. Oxaliplatin (OXA, 3 μM) and piplartine (PL, 10 μM) were used as the positive controls. Data are presented as the mean ± S.E.M. of three independent experiments performed in duplicate. Ten thousand events were evaluated per experiment and cellular debris was omitted from the analysis. ^*^ *p* < 0.05 compared with the negative control by ANOVA followed by Student Newman-Keuls test.

**Figure 9:**
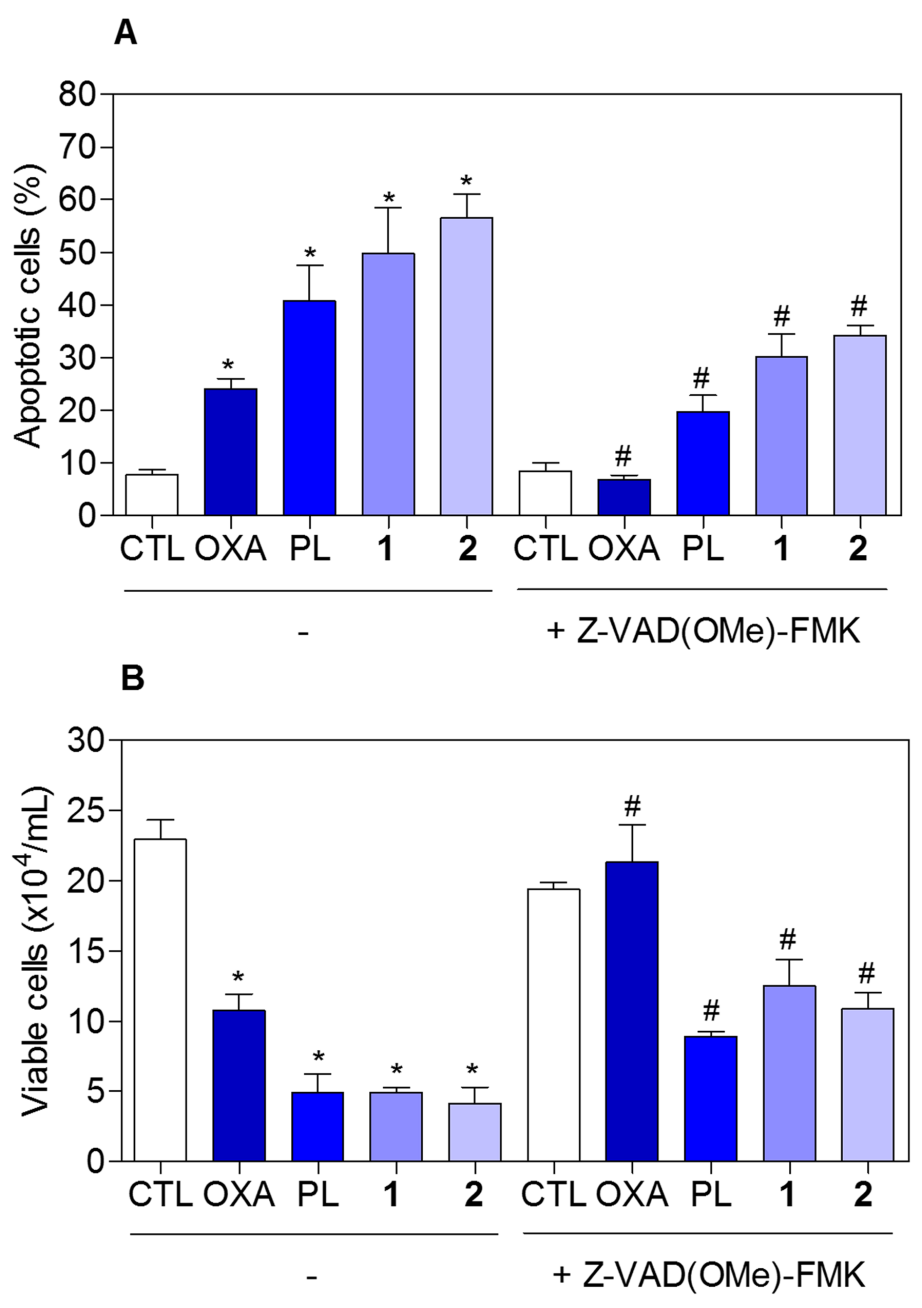
Effect of the pan-caspase inhibitor (Z-VAD(OMe)-FMK) in the apoptosis induced by piplartine-containing ruthenium complexes on HCT116 cells determined by flow cytometry using annexin V-FITC/PI staining **(A)** Quantification of apoptotic cells (early + late apoptotic cells) determined by flow cytometry using annexin V-FITC/PI staining. **(B)** Quantification of the cell viability determined by trypan blue staining. The cells were pre-treated for 2 h with 50 μM Z-VAD(OMe)-FMK, then incubated with the complexes in the established concentration (2.5 μM for complex **1** and 5 μM for complex **2**) for 48 h. The negative control (CTL) was treated with the vehicle (0.1% of a solution containing 70% sorbitol, 25% tween 80 and 5% water) used for diluting the compounds tested. Oxaliplatin (OXA, 3 μM) and piplartine (PL, 10 μM) were used as the positive controls. Data are presented as the mean ± S.E.M. of three independent experiments performed in duplicate. For flow cytometry analysis, 10,000 events were evaluated per experiment and cellular debris was omitted from the analysis. ^*^ *p* < 0.05 compared with the negative control by ANOVA followed by Student Newman-Keuls test. ^#^ *p* < 0.05 compared with the respective treatment without inhibitor by ANOVA followed by Student Newman-Keuls test.

**Figure 10:**
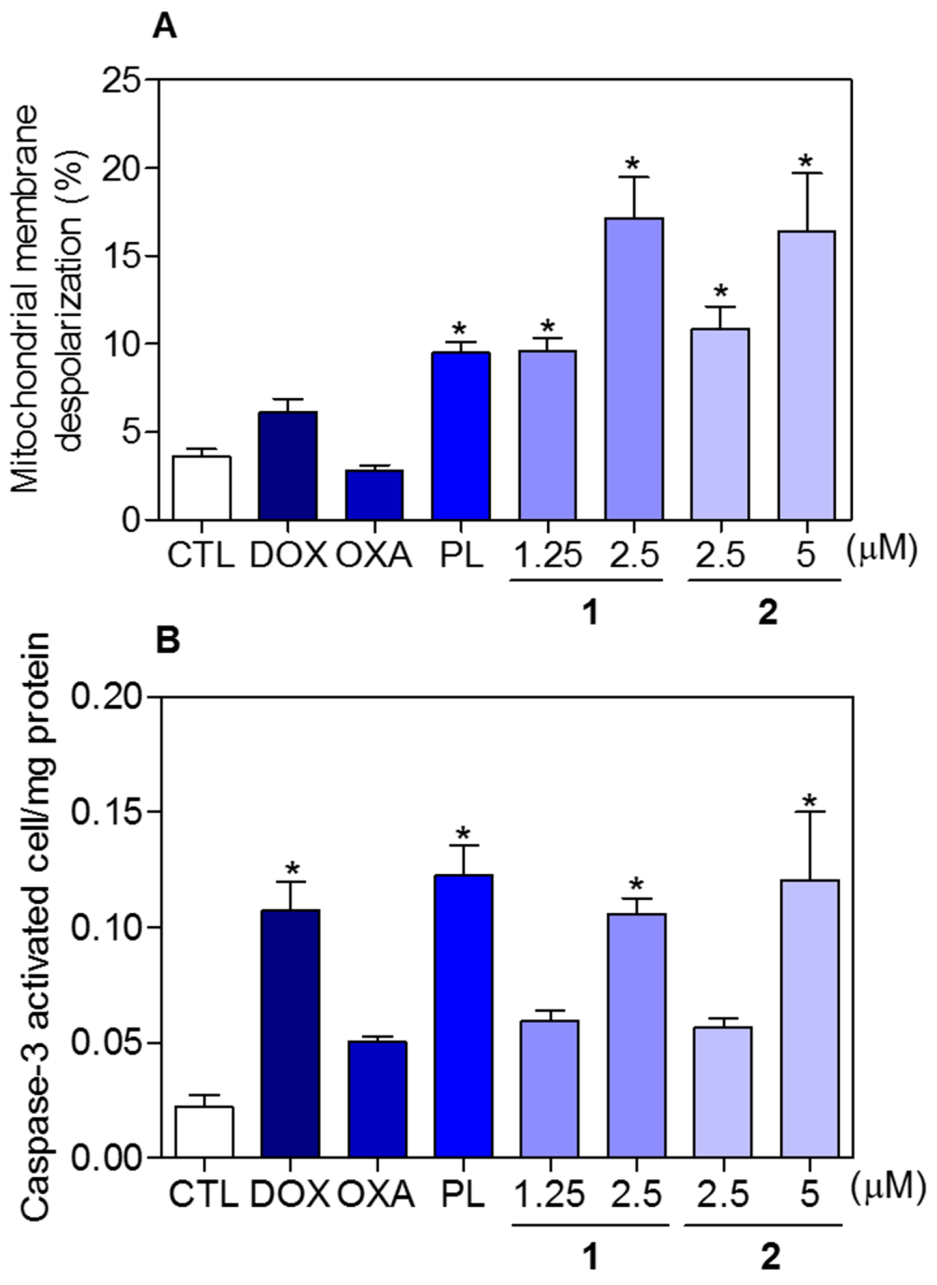
Effect of piplartine-containing ruthenium complexes in the mitochondrial membrane potential and caspase-3 activity on HCT116 cells **(A)** Mitochondrial membrane potential determined by flow cytometry using rhodamine 123 staining after 24h of incubation. **(B)** Caspase-3 activity determined by colorimetric assay after 48h of incubation. The negative control (CTL) was treated with the vehicle (0.1% of a solution containing 70% sorbitol, 25% tween 80 and 5% water) used for diluting the compounds tested. Doxorubicin (DOX, 1 μM), oxaliplatin (OXA, 3 μM) and piplartine (PL, 10 μM) were used as the positive controls. Data are presented as the mean ± S.E.M. of three independent experiments performed in duplicate. For flow cytometry analysis, 10,000 events were evaluated per experiment and cellular debris was omitted from the analysis. ^*^ *p* < 0.05 compared with the negative control by ANOVA followed by Student Newman-Keuls test.

To determine whether the complexes-induced cytotoxicity results from cell apoptosis induction, we performed annexin-V/PI double staining to identify the type of the cell death on HCT116 cells (Supplementary Figure 5 and Figure 8). The results clearly showed that both complexes increased the early and late apoptosis in a time- and concentration-dependent manners. None of complexes induced significant increase in necrotic cells. Moreover, co-treatment with a pan-caspase inhibitor, Z-Val-Ala-Asp-(OMe)-Fluoromethyl Ketone (Z-VAD(OMe)-FMK), prevented the complexes-induced increasing of cell death by apoptosis (Supplementary Figure 6 and Figure 9A). In addition, co-treatment with Z-VAD(OMe)-FMK also prevented the reduction of the number of viable cells assessed by trypan blue exclusion assay (Figure 9B). The complexes also induced mitochondrial depolarization on HCT116 cells, as measured by the incorporation of rhodamine 123 using flow cytometry (Figure 10A). Considering that the complexes were causing cell death by apoptosis, we next studied the caspase-3 activation of complexes-induced apoptosis using Asp-Glu-Val-Asp (DEVD)-pNA as the substrate. Both complexes induced activation of caspase-3 on HCT116 cells (Figure 10B). Piplartine also induced increasing in the early and late apoptosis, which was prevented by co-treatment with Z-VAD(OMe)-FMK. Mitochondrial depolarization and activation of caspase-3 were observed on piplartine-treated HCT116 cells.

The cytotoxic activity of the complexes on BAD gene knockout immortalized mouse embryonic fibroblast (BAD KO SV40 MEF) and its parental cell line wild-type immortalized mouse embryonic fibroblast (WT SV40 MEF) was also performed by alamar blue assay after 72 h incubation. The IC_50_ values for piplartine, complexes **1** and **2** were 4.0, 3.2 and 4.0 μM for BAD KO SV40 MEF cell line, while were 4.5, 2.4 and 6.0 μM for WT SV40 MEF cell line, suggesting that BAD gene is not essential for the cytotoxicity induced by piplartine or its ruthenium-based complexes. Doxorubicin presents IC_50_ values of 0.41 and 0.04 μM, while 5-fluorouracil presents IC_50_ values of 7.3 and 1.7 μM on BAD KO SV40 MEF and WT SV40 MEF cell lines, respectively.

### Piplartine-containing ruthenium complexes increase ROS levels on HCT116 cells

**Figure 11:**
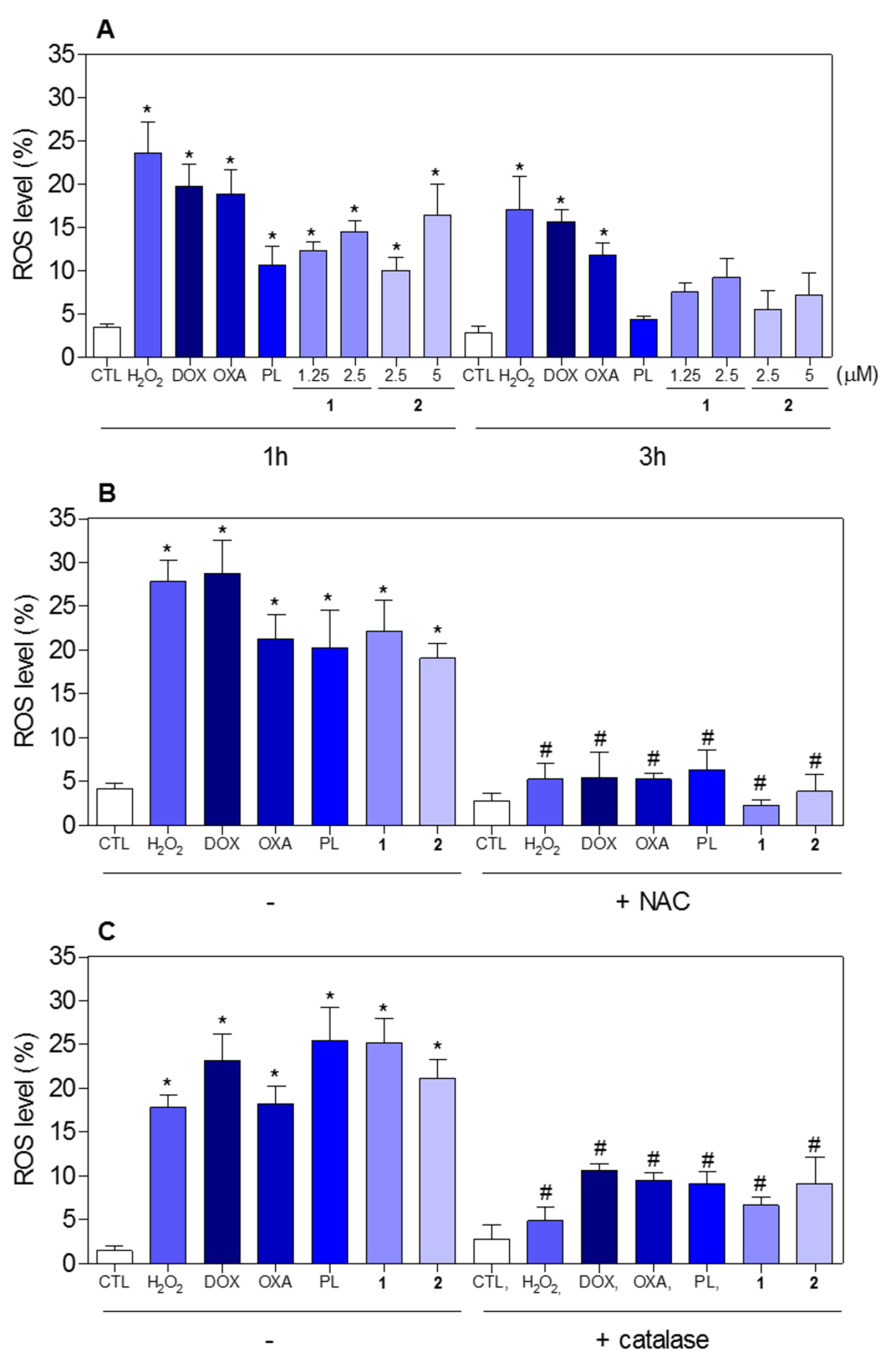
Effect of piplartine-containing ruthenium complexes in the levels of reactive oxygen species (ROS) of HCT116 cells and protection by NAC and catalase determined by flow cytometry using DCF-DA staining **(A)** ROS levels of HCT116 cells after 1 and 3h of incubation. **(B)** ROS levels of HCT116 cells pre-treated with the antioxidant NAC and, then treated with the complexes. **(C)** ROS levels of HCT116 cells pre-treated with the antioxidant catalase and, then treated with the complexes. For the protection assay, the cells were pre-treated for 1 h with 5 mM NAC or 2,000UI catalase, then incubated with the complexes in the established concentration (2.5 μM for complex **1** and 5 μM for complex **2**) for 1 h. The negative control (CTL) was treated with the vehicle (0.1% of a solution containing 70% sorbitol, 25% tween 80 and 5% water) used for diluting the compounds tested. Hydrogen peroxide (H_2_O_2_, 200 μM), doxorubicin (DOX, 1 μM), oxaliplatin (OXA, 3 μM) and piplartine (PL, 10 μM) were used as the positive controls. Data are presented as the mean ± S.E.M. of three independent experiments performed in duplicate or triplicate. Ten thousand events were evaluated per experiment and cellular debris was omitted from the analysis. ^*^ *p* < 0.05 compared with the negative control by ANOVA followed by Student Newman-Keuls test. ^#^ *p* < 0.05 compared with the respective treatment without inhibitor by ANOVA followed by Student Newman-Keuls test.

**Figure 12:**
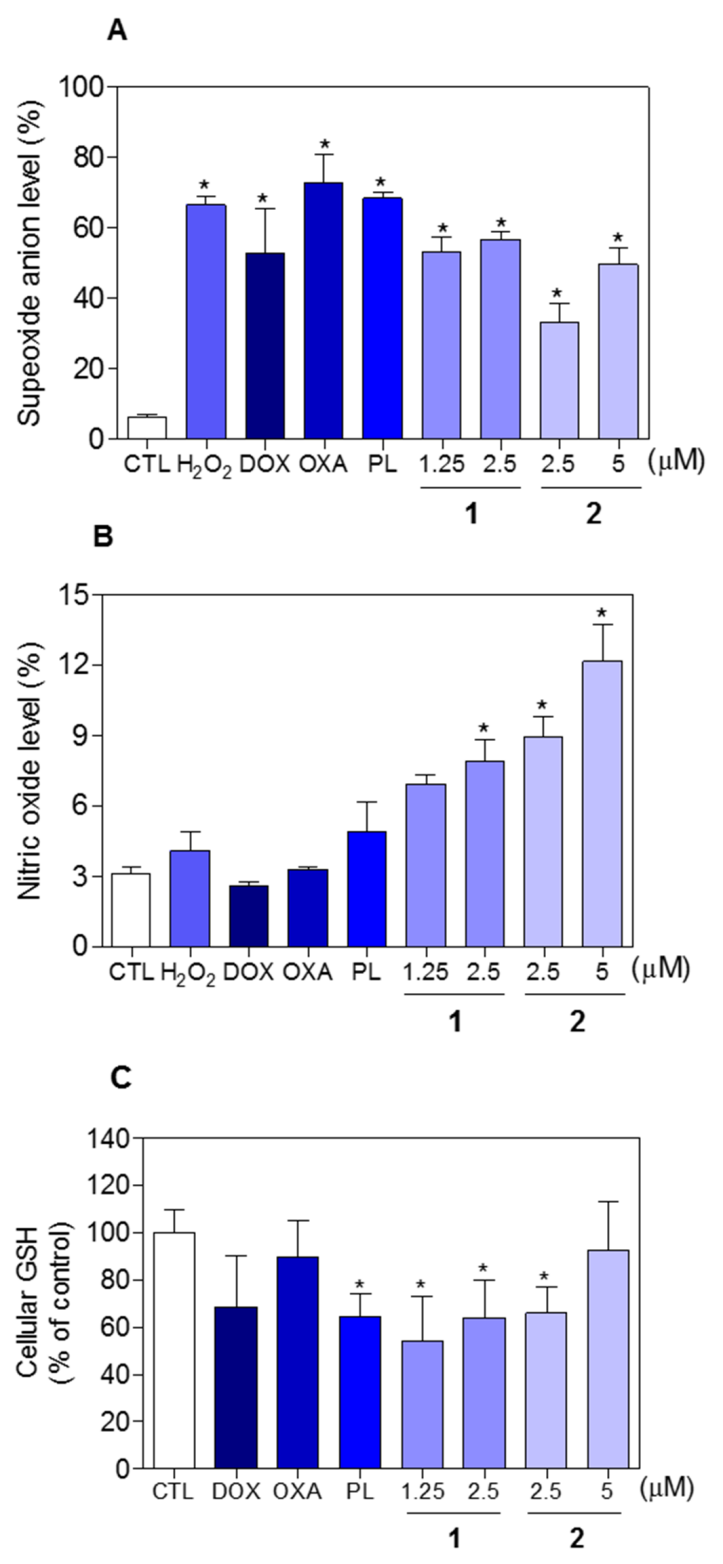
Effect of piplartine-containing ruthenium complexes in the levels of reactive oxygen species (ROS) and reduced glutathione (GSH) of HCT116 cells after 1 h incubation **(A)** Superoxide anion level of HCT116 cells determined by flow cytometry using hydroethidine staining. **(B)** Nitric oxide level of HCT116 cells determined by flow cytometry using DAF-FM diacetate staining. **(C)** GSH level of HCT116 cells determined by colorimetric assay. The negative control (CTL) was treated with the vehicle (0.1% of a solution containing 70% sorbitol, 25% tween 80 and 5% water) used for diluting the compounds tested. Hydrogen peroxide (H_2_O_2_, 200 μM), doxorubicin (DOX, 1 μM), oxaliplatin (OXA, 3 μM) and piplartine (PL, 10 μM) were used as the positive controls. Data are presented as the mean ± S.E.M. of three independent experiments performed in duplicate. For flow cytometry analysis, 10,000 events were evaluated per experiment and cellular debris was omitted from the analysis. ^*^ *p* < 0.05 compared with the negative control by ANOVA followed by Student Newman-Keuls test.

**Figure 13:**
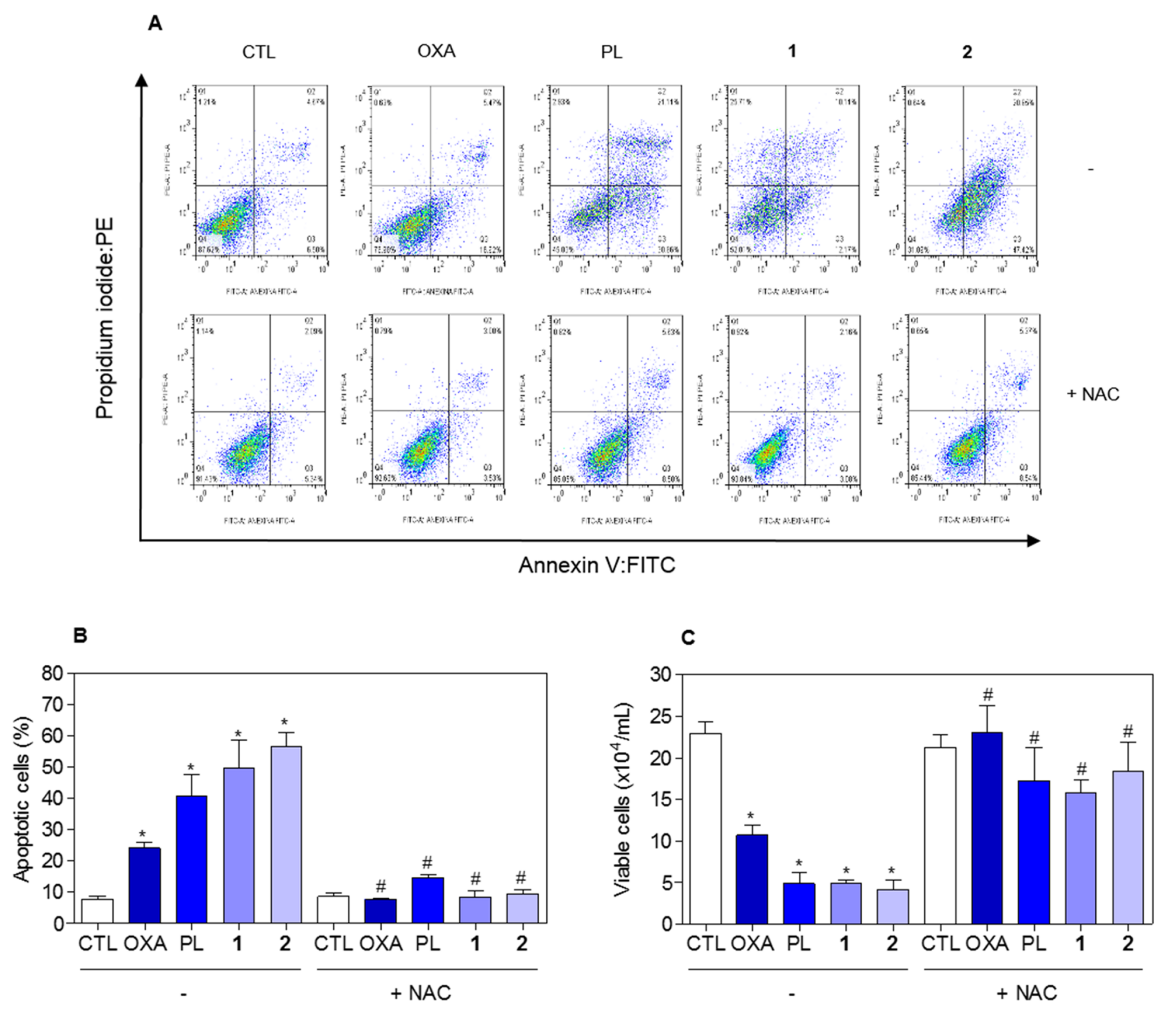
Effect of the antioxidant NAC in the apoptosis induced by piplartine-containing ruthenium complexes on HCT116 cells determined by flow cytometry using annexin V-FITC/PI staining **(A)** Representative flow cytometric dot plots showing the percentage of cells in viable, early apoptotic, late apoptotic and necrotic stages. **(B)** Quantification of apoptotic cells (early + late apoptotic cells) determined by flow cytometry using annexin V-FITC/PI staining. **(C)** Quantification of the cell viability determined by trypan blue staining. The cells were pre-treated for 1 h with 5 mM NAC, then incubated with the complexes in the established concentration (2.5 μM for complex **1** and 5 μM for complex **2**) for 48 h. The negative control (CTL) was treated with the vehicle (0.1% of a solution containing 70% sorbitol, 25% tween 80 and 5% water) used for diluting the compounds tested. Oxaliplatin (OXA, 3 μM) and piplartine (PL, 10 μM) were used as the positive controls. Data are presented as the mean ± S.E.M. of three independent experiments performed in duplicate. For flow cytometry analysis, 10,000 events were evaluated per experiment and cellular debris was omitted from the analysis. ^*^ *p* < 0.05 compared with the negative control by ANOVA followed by Student Newman-Keuls test. ^#^ *p* < 0.05 compared with the respective treatment without inhibitor by ANOVA followed by Student Newman-Keuls test.

The effect of piplartine-containing ruthenium complexes in intracellular ROS levels on HCT116 cells was determined by flow cytometry using the redox-sensitive fluorescent probe 2’-,7’-dichlorofluorescein diacetate (DCF-DA). Treatment with both complexes for 1 h caused a marked increase in ROS levels (Figure 11A). In addition, co-treatment with the antioxidant N-acetyl-L-cysteine (NAC) fully prevented the increase in the intracellular ROS level induced by the complexes (Figure 11B). Further, co-treatment with catalase, that induces decomposition of hydrogen peroxide, also prevented the complexes-induced increase in intracellular ROS levels, indicating the production of hydrogen peroxide induced by the complexes (Figure 11C). In a new set of experiments, we used the fluorescent probe hydroethidine and the fluorescent probe 4-amino-5-methylamino-2’-,7’-difluofluorescein diacetate (DAF-FM diacetate) for detect superoxide anion and nitric oxide, respectively. We found that superoxide anion (Figure 12A) and the nitric oxide (Figure 12B) were also among the ROS induced by the complexes on HCT116 cells. The reduced glutathione (GSH) levels were also decreased in complexes-treated cells (Figure 12C). Furthermore, co-treatment with NAC, prevented the increase of the cell death by apoptosis induced by the complexes (Figure 13A and 13B) and the reduction of the number of viable cells, as assessed by trypan blue exclusion assay (Figure 13C). Piplartine also induced increasing oxidative stress, which was prevented by co-treatment with NAC.

### Piplartine-containing ruthenium complexes do not induce DNA intercalation

Some ruthemium complexes can intercalate to DNA, thus we invertigated if the piplartine-containing ruthenium complexes are able to induce DNA intercalation. DNA intercalation was assessed by examining the ability of the complexes to displace ethidium bromide from calf thymus DNA (ctDNA). For this, the complexes were added to a DNA-ethidium bromide mixture to assess if the complexes compete with ethidium bromide and intercalate into DNA. If the complexes displace ethidium bromide from DNA, the fluorescence intensity of ethidium bromide decrease. None of complexes were able to decrease the ethidium bromide fluorescence, indicating that they are not strong DNA intercalators. Doxorubicin, a known DNA intercalator, significantly reduced fluorescence in this assay (data not shown).

### Piplartine-containing ruthenium complexes alter genic expression on HCT116 cells

The effect of piplartine-containing ruthenium complexes on the expression of 92 genes involved in different cellular mechanisms, including cell proliferation, cell cycle, apoptosis, oxidative stress, metastasis and angiogenesis, was detected after 12h of incubation on HCT116 cells by qPCR array using a 96-well plate TaqMan® Array Human Molecular Mechanisms of Cancer. A total of 31 genes were upregulated and 5 genes were downregulated after the treatment with piplartine-containing ruthenium complexes (Supplementary Table 2 and Table 4). Among them, the genes *CDKN2A* (RQ = 2.5), *FOS* (RQ = 11.9), *JUN* (RQ = 3.7), *NFKBIA* (RQ = 2.0), *PTEN* (RQ = 2.0) and *TP53* (RQ = 3.5) were upregulated in the cells treated with the complex **1**, while the gene *SHC1* (RQ = 0.4) was downregulated. After the treatment with the complex **2**, *FADD* (RQ = 74.1) and *JUN* (RQ = 2.3) were among the genes upregulated, while *CCND1* (RQ = 0.5) was among the genes downregulated. Doxorubicin and piplartine also induced change in different genes, incluing the upregulation of *FOS* (RQ = 11.8), *JUN* (RQ = 4.1), *NFKBIA* (RQ = 2.3) and *TP53* (RQ = 3.3) for doxorubicin and *FOS* (RQ = 31.0), *JUN* (RQ = 3.8) and *NFKBIA* (RQ = 2.0) for piplartine that also reduced the expression of the gene *SHC1* (RQ = 0.2).

**Table 4:** The effect of piplartine-containing ruthenium complexes on gene expression of HCT116 cells

Gene symbol	Description	RQ			
		DOX	PL	1	2
*BCL2*	BCL2, apoptosis regulator	3.1	1.1	2.4	0.8
*CCND1*	cyclin D1	1.1	0.7	1.2	0.5
*CDH1*	cadherin 1	7.1	2.5	3.7	1.1
*CDKN2A*	cyclin dependent kinase inhibitor 2A	1.8	1.6	2.5	1.2
*CYCS*	cytochrome c, somatic	1.0	1.0	2.0	1.0
*EGFR*	epidermal growth factor receptor	0.8	1.2	1.3	11,276.3
*FADD*	Fas associated via death domain	1.1	0.4	0.6	74.1
*FN1*	fibronectin 1	3.6	2.0	3.8	N.d.
*FOS*	Fos proto-oncogene, AP-1 transcription factor subunit	11.8	31.0	11.9	N.d.
*FYN*	FYN proto-oncogene, Src family tyrosine kinase	1.3	1.7	2.5	1.8
*FZD1*	frizzled class receptor 1	1.0	1.0	2.3	0.9
*GUSB*	glucuronidase beta	5.0	5.2	4.5	2.9
*IGF1R*	insulin like growth factor 1 receptor	1.4	2.1	2.3	0.7
*ITGA2B*	integrin subunit alpha 2b	4.5	3.0	3.1	0.1
*ITGAV*	integrin subunit alpha V	1.3	1.0	1.4	1,149.0
*JUN*	Jun proto-oncogene, AP-1 transcription factor subunit	4.1	3.8	3.7	2.3
*KDR*	kinase insert domain receptor	N.d.	8.3	4.4	1.4
*MAP2K1*	mitogen-activated protein kinase kinase 1	0.7	0.8	0.8	2,107.2
*MAP3K5*	mitogen-activated protein kinase kinase kinase 5	1.0	0.6	0.3	4.3
*MAPK3*	mitogen-activated protein kinase 3	2.8	1.7	2.0	1.9
*MAX*	MYC associated factor X	N.d.	3.1	3.6	0.9
*NFKBIA*	NF-κB inhibitor alpha	2.3	2.0	2.0	0.9
*PIK3R1*	phosphoinositide-3-kinase regulatory subunit 1	2.0	2.4	2.5	1.3
*PTEN*	phosphatase and tensin homolog	1.8	0.5	2.0	0.8
*PTK2*	protein tyrosine kinase 2	1.2	0.6	1.6	2.4
*PTK2B*	protein tyrosine kinase 2 beta	3.4	2.3	2.3	1.0
*RAC1*	ras-related C3 botulinum toxin substrate 1 (rho family, small GTP binding protein Rac1)	0.9	0.7	1.1	2.7
*RAF1*	Raf-1 proto-oncogene, serine/threonine kinase	2.0	0.6	2.0	0.9
*SHC1*	SHC adaptor protein 1	0.6	0.2	0.4	N.d.
*SMAD4*	SMAD family member 4	1.0	0.9	1.4	2.1
*SOS1*	SOS Ras/Rac guanine nucleotide exchange factor 1	0.7	0.9	1.2	0.2
*SPP1*	secreted phosphoprotein 1	1.2	1.3	2.5	10.1
*TGFBR2*	transforming growth factor beta receptor 2	0.9	1.2	2.4	1.1
*TP53*	tumor protein p53	3.3	1.2	3.5	1.5

HCT116 cells were treated with 2.5 μM of complex **1** and 5 μM of complex **2** for 12 h. The negative control was treated with the vehicle (0.1% of a solution containing 70% sorbitol, 25% tween 80 and 5% water) used for diluting the compounds tested. Doxorubicin (DOX, 1 μM) and piplartine (PL, 10 μM) were used as the positive controls. After treatment, total RNA was isolated and reverse transcribed. Gene expression was detected using the 96-well plate TaqMan® Array Human Molecular Mechanisms of Cancer. *GAPDH*, *18S* and *HPRT1* genes were used as endogenous gene for normalization. Values represent the relative quantitation (RQ) compared with the calibrator (cells treated with the negative control, RQ = 1.0). The genes were considered to be upregulated if RQ ≥ 2 and were considered to be downregulated if RQ ≤ 0.5. N.d. Not determinated.

Interestingly, complex **2** increased substantially the expression of *EGFR* gene (RQ = 11,276.3). The activation of this class of receptor leads to cell proliferation, which is target of the class of drugs tyrosine kinase inhibitors. Although this being an unexpected effect, the increase of *EGFR* gene expression on cancer cells can take it more susceptible to tyrosine kinase inhibitors, indicating a potential synergic effect of complex 2 with this class of drugs.

## DISCUSSION

Piplartine is a well characterizated cytotoxic agent, able to induce ROS selectively in different cancer cells, leading to cell death by apoptosis [3–18]. Many piplartine analogs have been synthesized and evaluated against cancer cells; however, this class of compounds has never been employed as ligand for composition of metal complexes. In this present paper, two novel piplartine-containing ruthenium complexes were designed, synthesized and evaluated for their cytotoxic potential.

The ruthenium complexes produced in our study were tested against cancer cells, showing potent cytotoxic activity. Other studies have shown that complexes of ruthenium with different ligands can induce cytotoxic activity in micromolar or molar range against several cancer cell lines [44–46]. Moreover, piplartine is able to kill cancer cells of different histological types, including hematological, colon, melanocyte, lung, breast, central nervous system, pancreatic, nasopharyngeal, osseous, bladder, renal, and prostate in micromolar range [10, 12, 18]. In the present work, both piplartine-containing ruthenium complexes showed cytotoxic activity up to 12 fold higher than metal-free piplartine. In 3D culture that mimics the *in vivo* cancer tissue, the piplartine-containing ruthenium complexes were also more potent in inducing citotoxicity than metal-free piplartine.

Regarding their mechanism of action, the piplartine-containing ruthenium complexes were shown to induce caspase-dependent and mitochondrial intrinsic apoptosis on HCT116 cells as observed by cell shrinkage, internucleosomal DNA fragmentation without alter the cell membrane permeabilization, externalization of phosphatidylserine, loss of mitochondrial transmembrane potential and marked activation of caspase-3. In addition, the apoptosis induction and the reduction of the number of viable cells were prevented by pre-treatment with Z-VAD(OMe)-FMK, a pan-caspases inhibitor.

Several studies have shown that piplartine induces cell death by triggering apoptosis pathway. Piplartine was shown to reduce the number of viable cells and induces cell apoptosis on HCT116 cells by JNK signaling pathway [23], and induces apoptosis and autophagy through modulation of the PI3K/Akt/mTOR pathway in human lung cancer cells [22]. It can also induce apoptotic cell death and suppress the DNA binding activity of NF-κB in a concentration-dependent manner in non-small cell lung cancer cells [15]. The ruthenium complexes can also induce apoptosis of cancer cells through pathways mediated by death receptor, mitochondria and/or endoplasmic reticulum stress. The binding behavior of ruthenium complexes with DNA, especially with Gquadruplex DNA may play a important role in the DNA damage of cancer cells [44–51]. In our study, however, the piplartine-containing ruthenium complexes failed to induce DNA intercalation, despite being able to induce cell death by apoptosis.

As mentioned above, piplartine induces ROS increase and apoptotic cell death on cancer cells [10, 12]. Additionally, piplartine induces a lethal endoplasmic reticulum stress and mitochondrial dysfunction by inhibiting TrxR1 activity and increasing intracellular ROS levels [25]. It is well-known that ruthenium (II) complexes can induce apoptosis through ROS-mediated pathway in cancer cells [44, 46]. Therefore, since ruthenium complexes and piplartine have been described as ROS inductor agents, we measured the ROS level in cells treated with piplartine-containing ruthenium complexes. In fact, piplartine-containing ruthenium complexes were also able to increse ROS level, assigned to nitric oxide, superoxide anion and hydrogen peroxide. GSH levels were also decreased in complexes-treated cells. Interestingly, the pre-treatment with the antioxidant NAC prevented the apoptosis induction and the reduction of the number of viable cells caused by both piplartine-containing ruthenium complexes, indicating ROS-mediated apoptosis cell death pathway.

The molecular mechanism underlying the cytotoxic effect of piplartine-containing ruthenium complexes was assessed at the level of mRNA expression of several genes. In special, it was observed the increasing of the expression of *CDKN2A*, *FOS*, *JUN*, *NFKBIA*, *TP53* and *FADD*, and reduction of the expression of *SHC1* and *CCND1*. *CCND1* (cyclin D1) gene is involved in the cell cycle G_1_/S transition. Moreover, *CDKN2A* (cyclin dependent kinase inhibitor 2A) gene was upregulated. Jyothi et al. [16] demonstrated that piplartine is able to reduce the protein levels of cyclin D1 in the mouse embryonal carcinoma cell line. Ruthenium benzimidazole complex also have been reported as a cyclin D1 inhibitor [44]. The protein encoded by *FADD* (Fas associated via death domain) gene mediates cell apoptotic signals. *FOS* (Fos proto-oncogene) gene encode proteins that can dimerize with proteins of the JUN (Jun proto-oncogene, AP-1 transcription factor subunit) family leading to the formation of the transcription factor complex AP-1, which is involved in the transcription of some pro-apoptotic proteins [52, 53]. Furthermore, JNK-AP-1 pathway is involved in the increase of the expression of pro-apoptotic genes, including Fas-L and Bak [54]. In fact, several apoptotic-related genes have been also associated with the effect of ruthenium complexes and/or piplartine on cancer cells [22, 23, 44, 46, 48, 51]. In addition, Li et al. [23] demostrated that piplartine can induce apoptosis on HCT116 cells by JNK signaling pathway. In this present work, we also observed that piplartine-containing ruthenium complexes are able to induce caspase-dependent and mitochondrial intrinsic apoptosis on HCT116 cells. *NFKBIA* (NF-κB inhibitor alpha) gene encodes a member of NF-κB inhibitor family. As mentioned above, piplartine can lead to apoptotic cell death by NF-κB inhibition in non-small cell lung cancer cells [15]. The *TP53* (tumor protein p53) gene encodes a tumor suppressor protein. Interestingly, cell death induced by piplartine has been observed by both dependent and independent p53 signaling [12]. Thiazolo arene ruthenium complexes also can induce cell death by p53 signaling in cisplatin-sensitive and cisplatin resistant ovarian cancer cells [55]. The gene *SHC1* (SHC adaptor protein 1) belong to the mammalian Shc family, composed by p52Shc, p46Shc and p66Shc isoforms. Curiously, p66Shc has antioxidant action on the cytoplasm. As mentioned above, piplartine and several ruthenium complexes have been reported as ROS inducers [10, 12, 46]. Here, we also observed that piplartine-containing ruthenium complexes induce ROS.

The similarity between the mechanism of action of piplartine and its ruthenium-based complexes suggests that the complexes can be acting as carriers of piplartine, increasing its bioavailability mainly due to the lability of the coordinated molecule from the complexes, in the presence of water. The ability of release ligands through the aquaction reaction has already been reported to other ruthenium compounds, such as NAMI/NAMI-A complexes [31, 56].

In conclusion, the novel piplartine-containing ruthenium complexes **1** and **2** display more potent cytotoxicity than metal-free piplartine against different cancer cell lines and are able to induce caspase-dependent and mitochondrial intrinsic apoptosis on HCT116 cells by ROS-mediated pathway. No DNA intercalation was observed with piplartine-containing ruthenium complexes. Additionally, the level of mRNA expression of several genes, including cell proliferation, cell cycle, apoptosis and oxidative stress were regulated under treatment. Thus, the novel piplartine-containing ruthenium complexes are more potent than metal-free piplartine, and, similar piplartine, target the oxidative stress.

## MATERIALS AND METHODS

### Synthesis of novel piplartine-containing ruthenium complexes

#### General

All procedures involving solutions of the complexes were performed under inert atmosphere (argon). The solvents used in the manipulations were purified by standard methods. The RuCl_3_.3H_2_O, 1,4-bis(diphenylphosphino)butane (dppb) 1,1-bis(diphenyl phosphino)ferrocene (dppf), 2,2’-bipyridine (bipy), were purchased from Sigma-Aldrich (Sigma-Aldrich Co., Saint Louis, MO, USA) and the piplartine was purchased from Cayman Chemical Company (Cayman Chemical, Ann Arbor, MI, USA). The precursors *cis*-[RuCl_2_(dppb)(bipy)] and *cis*-[RuCl_2_(dppf)(bipy)], were prepared according to published procedures [34, 39].

The vibrational spectroscopy in the infrared region was recorded on a FT-IR Bomem Michelson 102 spectrometer in the 4000 – 350 cm^-1^ region using KBr pellets. Conductance data were performed in dry acetone, using a Micronal model B-330 connected to a platinum electrode, with 0.089/cm constant cell, using 1 mM solutions of the complexes, at room temperature. The elemental analyses were performed with EA 1108 CHNS microanalyser (Fisons Instruments), in the Microanalytical Laboratory of the Chemistry Department of the Federal University of São Carlos. Electrochemical experiments (cyclic and pulse voltammetry) were carried out at 25 °C in CH_2_Cl_2_ containing 0.10 M Bu_4_NClO_4_ (tetrabutylammonium perchlorate, TBAP), recorded with a Bioanalytical Systems Inc electrochemical analyzer, model BAS-100B/W. The working and auxiliary electrodes were stationary platinum; a system Ag/AgCl in Luggin capillary probe was used as a reference electrode. Under these conditions, the ferrocene couple (Fc^+^/Fc) is oxidized at 0.43 V. The NMR experiments (^1^H, ^13^C{^1^H, ^31^P{^1^H}, ^1^H-^1^H gCOSY, ^1^H-^13^C{^1^H} gHSQC and DEPT-135) were recorded on a Bruker DRX 400 Ultrashield™ (400.132 MHz for hydrogen frequency, 100.623 MHz for carbon frequency and 161.976 MHz for phosphorous frequency), referenced with TMS (tetramethylsilane). In the ^1^H, ^13^C{^1^H}, ^1^H-^1^H gCOSY, ^1^H-^13^C{^1^H} gHSQC and DEPT-135 experiments, 25 mg of final metal complexes were dissolved in deuterated acetone (DMSO-*d*_*6*_, Cambridge Isotope Laboratories, Inc., USA). ^31^P{^1^H} stability experiments are reported in relation to H_3_PO_4_ (85% v/v), using a capillary containing D_2_O.

#### [Ru(piplartine)(dppf)(bipy)](PF_6_)_2_ (1)

To a Schlenk flask containing 10 mL of dichlorometane and 10 mL of acetone, the *cis-*[RuCl_2_(dppf)(bipy)] (0.066 mmol) and piplartine (0.099 mmol) were added. After the complete solubilization of the solids, 0.165 mmol of AgPF_6_ was added, forming, immediately, a white precipitate. The solution was kept at room temperature and darkness, under an inert atmosphere and under stirring for 1 h. Next, the solvent was reduced to 2 mL, and 10 mL of CH_2_Cl_2_ was added, to form a white powder (AgCl), which was filtered off. The filtered solution was concentrated to 3 mL and hexane (10 mL) was added to precipitate an orange powder, which was filtered off, washed with hexane, diethyl ether, and dried under vacuum. Yield: 70 mg (87%). Anal. Calc. for [C_61_H_55_FeN_3_O_5_P_2_Ru](PF_6_)_2_: exp. (calc) C, 51.76 (51.64); H, 3.75 (3.91); N, 2.65 (2.96) %. Molar conductance (S cm^2^ mol^-1^, acetone) 193.9. IR (cm^-1^): (nCH) 3093, (nCH_2_) 2942, (nCH) 2845; (nC=O) 1650; (nC=N) 1598; (nC=C and νC=N) 1544, 1502, 1434, 1421, 1332, 1305; (nC-O) 1122; (nP-C) 1093; (n_ring_) 1000; (nP-F) 844; (nC-H-cp) 750; (nP-CH) 636; (δP-F) 557; (nRu-P) 520 and 495; (nRu-O + nFe-cp) 474; (nRu-N) 428. ^31^P{^1^H} NMR (162 MHz, d_6_-acetone, 298 K): δ (ppm) (d, 39.1; 39.3 / 43.1; 45.9, ^2^*J* = 29.6 / 30.3 Hz); 1H NMR (400 MHz, d_6_-acetone, 298 K): δ (ppm): 9.00, 8.93 (1H, d, bipy); 8.56, 8.50 (2H, br. s, bipy); 8.30 (0.5H, s. bipy) 8.14 (2H, m, bipy); 7.76–6.87 (28H, m, Ho, Hm and Hp of dppf, 2.5H bipy, 2H, H_aromatic_ of piplartine, 3H H_double_ piplartine); 6.74, 6.11 (1H, d, H_double_ piplartine); 5.20 – 4.42 (8H, m, cp of ferrocene); 4.02 - 3.95 (5H, s+m) (CH_3_ + CH_2_ of piplartine), 3.88 (1.5H, s), 3.80 (3H, s), 3.76 (1.5H, s) (CH_3_ of piplartine); and 2.55 – 2.44 (2H, m, CH_2_ piplartine). ^13^C{^1^H} NMR (125.74 MHz, d_6_-acetone, 298 K): δ (ppm) 174.22, 172.29, 171.92, 170.38 (C=O); 163.50–150.64 (C-Bipy, C-piplartine); 143.53 – 108.16 (C-dppb, C-Bipy, C-piplartine); 81.30 – 73.29 (C-cp ferrocene), 60.93 – 56.83 (CH_3_ of piplartine) and 48.70 – 48.40, 25.15 – 24.95 (CH_2_ of piplartine).

#### [Ru(piplartine)(dppb)(bipy)](PF_6_)_2_ (2)

For the synthesis of this complex the same procedure of complex **1** was used. Yield: 75 mg (88%). Anal. Calc. for [C_55_H_55_N_3_O_5_P_2_Ru](PF_6_)_2_: exp. (calc) C, 50.85 (51.17); H, 4.45 (4.29); N, 3.04 (3.25) %. Molar conductance (S cm^2^ mol^-1^, acetone) 193.9. IR (cm^-1^): (νCH) 3061, (νCH_2_) 2936, (νCH) 2862; (νC=O) 1651; (νC=N) 1600; (νC=C and νC=N) 1579, 1501, 1463, 1435, 1421, 1328, 1304; (νC-O) 1122; (νP-C) 1096; (ν_ring_) 1002; (νP-F) 842; (νC-H-cp) 743; (νP-CH_2_) 698; (δP-F) 557; (νRu-P) 520 and 489; (νRu-O) 491; (νRu-N) 447. ^31^P{^1^H} NMR (162 MHz, d_6_-acetone, 298 K): δ (ppm) (d, 39.4; 42.3 / 41.0; 41.7, ^2^*J* = 32.6 / 34.9 Hz); 1H NMR (400 MHz, d_6_-acetone, 298 K): δ (ppm): 8.79, 8.69 (1H, d, bipy); 8.71, 8.60 (1H, br. s, bipy); 8.38 – 8.33 (1H, d. bipy); 8.25 (1H, d bipy); 7.06 (1H, s, H_arom_ of piplartine); 6.93 (1H, s, H_arom_ of piplartine); 8.22 – 6.18 (29H, m, Ho, Hm and Hp of dppf, bipy, H_double_ of piplartine); 3.94 (3H, s), 3.89 (1.5H, s), 3.81 (3H, s), 3.79 (1.5H, s) (CH_3_ of piplartine); 4.12 – 4.01, 3.59 – 3.45, 3,18 – 2.98, 2,75 – 2.14 and 1.58 (12H, m, CH_2_ of dppb and CH_2_ of piplartine). ^13^C{^1^H} NMR (125.74 MHz, d_6_-acetone, 298 K): δ (ppm) 173.76, 172.05, 171.69, 170.68 (C=O); 162.35–150.09 (C-Bipy, C-piplartine); 143.51 – 108.11, (C-dppb, C-Bipy, C-piplartine); 60.80 – 56.77 (CH_3_ of piplartine) and 48.52 – 48.14 (CH_2_ of piplartine) and 28.90 – 23.31 (CH_2_ of piplartine and CH_2_ of dppb).

### Cells

HCT116 (human colon carcinoma), HepG2 (human hepatocellular carcinoma), HSC-3 (human oral squamous cell carcinoma), SCC-4 (human oral squamous cell carcinoma), SCC-9 (human oral squamous cell carcinoma), HL-60 (human promyelocytic leukemia), K-562 (human chronic myelogenous leukemia), B16-F10 (murine melanoma), MRC-5 (human lung fibroblast), WT SV40 MEF (wild-type immortalized mouse embryonic fibroblast) and BAD KO SV40 MEF (BAD gene knockout immortalized mouse embryonic fibroblast) cell lines were obtained from the American Type Culture Collection (ATCC, Manassas, VA, USA) and cultured in RPMI-1640 medium (Gibco-BRL, Gaithersburg, MD, USA) with 10% fetal bovine serum (Life, Carlsbad, CA, USA), 2 mM L-glutamine (Vetec Química Fina, Duque de Caxias, RJ, Brazil) and 50 μg/mL gentamycin (Life, Carlsbad, CA, USA). Adherent cells were collected by treatment with 0.25% trypsin EDTA solution (Gibco-BRL, Gaithersburg, MD, USA). All cell lines were cultured in flasks at 37°C in 5% CO_2_ and sub-cultured every 3-4 days to maintain exponential growth. All cell lines were tested for mycoplasma using a mycoplasma stain kit (Sigma-Aldrich Co., Saint Louis, MO, USA) to validate the use of cells free from contamination.

Heparinized blood from 20-35 year-old, non-smoker healthy donors who had not taken any drugs for at least 15 days prior to sampling was collected, and the peripheral blood mononuclear cells (PBMCs) were isolated using a ficoll density gradient in a GE Ficoll-Paque Plus (GE Healthcare Bio-Sciences AB, Sweden). PBMCs were washed and resuspended at a concentration of 3 x 10^5^ cells/mL in RPMI 1640 medium with 20% fetal bovine serum, 2 mM glutamine and 50 μg/mL gentamycin at 37°C with 5% CO_2_. Concanavalin A (ConA, Sigma-Aldrich Co., Saint Louis, MO, USA) was used as a mitogen to trigger cell division in T-lymphocytes. ConA (10 μg/mL) was added at the beginning of culture, and the cells were treated with the test compounds after 24 h. Cell viability was examined using trypan blue exclusion assay for all experiments. Over 90% of the cells were viable at the beginning of the culture. The Research Ethics Committee of the Oswaldo Cruz Foundation (Salvador, Bahia, Brazil) approved the experimental protocol (# 031019/2013). All participants signed a written informed consent to participate in the study.

### Cytotoxic activity assay

Cell viability was quantified using the alamar blue assay, according to Ahmed et al. [57]. Cells were inserted in 96-well plates for all experiments (7 x 10^4^ cells/mL for adherent cells or 3 x 10^5^ cells/mL for suspended cells in 100 μL of medium). After 24 h, the complexes (in a range of eight different concentrations from 0.19 to 25 μg/mL) were dissolved in 0.1% of a solution containing 70% sorbitol, 25% tween 80 and 5% water and the solution was added to each well and incubated for 72h. Negative controls received the vehicle that was used for diluting the compounds tested. Doxorubicin (purity ≥ 95%, doxorubicin hydrochloride, Laboratory IMA S.A.I.C., Buenos Aires, Argentina), oxaliplatin (Sigma-Aldrich Co., Saint Louis, MO, USA) and piplartine (purity > 98%, Cayman Chemical, Ann Arbor, MI, USA) were used as the positive controls. Four (for cell lines) or 24 h (for PBMCs) before the end of incubation, 20 μL of a stock solution (0.312 mg/mL) of the alamar blue (resazurin, Sigma-Aldrich Co., Saint Louis, MO, USA) were added to each well. Absorbance at 570 nm and 600 nm was measured using the SpectraMax 190 Microplate Reader (Molecular Devices, Sunnyvale, CA, EUA), and the drug effect was quantified as the percentage of control absorbance.

### 3D multicellular spheroids culture

HCT116 cells were cultivated in 3D multicellular spheroids. Briefly, 100 μL of a solution of cells (0.5 x 10^6^ cells/mL) were inserted in 96-well plate with a cell-repellent surface (Greiner Bio-One, Kremsmünster, Austria) and cultured in RPMI 1640 medium with 10% fetal bovine serum, 2 mM glutamine, 3% matrigel (BD Biosciences, San Jose, CA, EUA) and 50 μg/mL gentamycin at 37°C with 5% CO_2_. Spheroids with stable structures and diameters had formed after three days. Then, the complexes (in a range of eight different concentrations varying of 0.19 to 25 μg/mL) were dissolved in 0.1% of a solution containing 70% sorbitol, 25% tween 80 and 5% water and the solution was added to each well and incubated for 72h. Negative control received the vehicle that was used for diluting the compounds tested. In the end of the experiment, morphological changes were examined by light microscopy (Olympus BX41, Tokyo, Japan) using Image-Pro software (Media Cybernetics, Inc. Silver Spring, USA) and the cell viability was determined by alamar blue assay as described above.

To investigate the mechanisms involved in cytotoxic action of the complexes, a new set of experiments was performed. In all experiments, 2 mL of a HCT116 cell solution (7 x 10^4^ cells/mL) was inserted in 24-well plates and incubated overnight to allow the cells to adhere to the plate surface. The cells were then treated for 24 and/or 48 h with the complexes (1.25 and 2.5 μM for complex **1** and 2.5 and 5 μM for complex **2**). The negative control was treated with the vehicle (0.1% of a solution containing 70% sorbitol, 25% tween 80 and 5% water) used for diluting the compounds tested. The stability of the complexes in the vehicle (70% sorbitol, 25% tween 80 and 5% water) was evaluated, which the complexes remained stable. Doxorubicin (1 μM), oxaliplatin (3 μM) and piplartine (10 μM) were used as the positive controls. Cell viability was assessed using trypan blue exclusion assay for all experiments.

### Morphological analysis

To evaluate alterations in morphology, cells were cultured under coverslip and stained with may-grunwald-giemsa. Morphological changes were examined by light microscopy using Image-Pro software. In addition, light scattering features was determined by flow cytometry on a BD LSRFortessa cytometer using the BD FACSDiva Software (BD Biosciences, San Jose, CA, EUA) and Flowjo Software 10 (Flowjo LCC, Ashland, OR, EUA). Ten thousand events were evaluated per experiment and cellular debris was omitted from the analysis.

### Internucleosomal DNA fragmentation and cell cycle distribution

Cells were harvested in a permeabilization solution containing 0.1% triton X-100 (Sigma Chemical Co. St Louis, MO, USA), 2 μg/mL propidium iodide (Sigma Chemical Co. St Louis, MO, USA), 0.1% sodium citrate and 100 μg/mL RNAse (Sigma Chemical Co. St Louis, MO, USA) and incubated in the dark for 15 min at room temperature [58]. Finally, cell fluorescence was measured by flow cytometry as described above.

### Annexin-V/PI staining assay

For apoptosis detection, we used a FITC Annexin V Apoptosis Detection Kit I (BD Biosciences, San Jose, CA, EUA) and the analysis were performed according to the manufacturer’s instructions. Briefly, the cells treated were collected after the indicated time, washed twice with cold saline and resuspended in 300 μL binding buffer. Then, it was added 5 μL of Annexin V-FITC staining buffer and 5 μL of propidium iodide. The mixture was incubated in the dark at 37°C for 15 min. Cell fluorescence was measured by flow cytometry as described above.

The protection assay using the pan-caspase inhibitor, Z-VAD(OMe)-FMK (Cayman Chemical, Ann Arbor, MI, USA), was performed. In brief, the cells were pre-treated for 2 h with 50 μM Z-VAD(OMe)-FMK, then incubated with the complexes in the established concentration for 48 h. The cells were then trypsinized and the FITC Annexin V Apoptosis Detection assay and trypan blue exclusion assay were conducted as described above.

### Measurement of the mitochondrial transmembrane potential

Mitochondrial transmembrane potential was determined by the retention of the dye rhodamine 123 [59]. Cells were incubated with rhodamine 123 (5 μg/mL, Sigma-Aldrich Co., Saint Louis, MO, USA) at 37 °C for 15 min in the dark and washed with saline. The cells were then incubated again in saline at 37 °C for 30 min in the dark and cell fluorescence was determined by flow cytometry as described above.

### Caspase-3 activation assay

A caspase-3 colorimetric assay kit (Sigma-Aldrich Co., Saint Louis, MO, USA) was used to investigate caspase-3 activation on complexes-treated HCT116 cells based on the cleavage of DEVD-pNA and the analysis was performed according to the manufacturer’s instructions. Briefly, cells were lysed by incubation with cell lysis buffer on ice for 10 min and then centrifuged. Enzyme reactions were carried out in a 96-well flat-bottom microplate. To each reaction mixture, 5 μL cell lysate was added. Absorbance at 405 nm was measured using the SpectraMax 190 Microplate Reader (Molecular Devices, Sunnyvale, CA, EUA). The results were expressed as specific activity (IU/mg protein) of caspase-3.

### Measurement of cellular reactive oxygen species levels

The levels of ROS were measured according to previously described [60] using DCF-DA (Sigma-Aldrich Co., Saint Louis, MO, USA). In brief, cells were treated with the complexes for 1 and 3 h. Then, the cells were collected, washed with saline and resuspended in FACS tubes with saline containing 5 μM DCF-DA for 30 min. Finally, the cells were washed with saline and the cell fluorescence was determined by flow cytometry as described above.

The protection assay using the antioxidant NAC (Sigma-Aldrich Co., Saint Louis, MO, USA) or catalase (Sigma-Aldrich Co., Saint Louis, MO, USA) was performed. In brief, the cells were pre-treated for 1 h with 5 mM NAC or 2,000 UI catalase, then incubated with the complexes in the established concentration for 1 h. The cells were then trypsinized and the ROS levels were measured as described above. In a new set of experiments, the cells were pre-treated for 1 h with 5 mM NAC, then incubated with the complexes in the established concentration for 48 h. The cells were then trypsinized and the FITC Annexin V Apoptosis Detection assay and trypan blue exclusion assay were conducted as described above.

### Measurement of cellular superoxide anion level

Hydroethidine (Sigma-Aldrich Co., Saint Louis, MO, USA) was used to detect cellular superoxide levels after 1 h of treatment with the complexes [61]. The cells were labeled with 10 μM of hydroethidine for 30 min. Finally, the cells were washed with saline and the cell fluorescence was determined by flow cytometry as described above.

### Measurement of nitric oxide production

Nitric oxide generation was detected with DAF-FM diacetate (Molecular Probes, Eugene, OR, USA) [62]. The cells were labeled with 3 μM of DAF-FM diacetate for 60 minutes at 37°C. Following staining cells were washed with saline and incubated for an additional 15 minutes at 37°C to allow for complete deesterification of the intracellular diacatates. Then the nitric oxide radical was measured by flow cytometry as described above.

### Measurement of cellular GSH level

A quantification kit for reduced glutathione (Sigma-Aldrich Co., Saint Louis, MO, USA) was used to investigate cellular GSH level on complexes-treated HCT116 cells and the analysis was performed according to the manufacturer’s instructions. Absorbance at 405 nm was measured using the SpectraMax 190 Microplate Reader (Molecular Devices, Sunnyvale, CA, EUA).

### DNA intercalation assay

DNA intercalation was assessed by examining the ability of the complexes to displace ethidium bromide from ctDNA (Sigma-Aldrich Co., Saint Louis, MO, USA) [63]. Sexplicate assays (100 μL) were conducted in 96-well plates and contained 15 μg/mL ctDNA, 1.5 μM ethidium bromide and 5, 10 and 20 μM of each complexes in saline solution. The vehicle (0.1% of a solution containing 70% sorbitol, 25% tween 80 and 5% water) used for diluting the compounds tested was also used as the negative control. Doxorubicin (10 μM) was used as the positive control. Fluorescence was measured using excitation and emission wavelengths of 320 nm and 600 nm, respectively using the spectraMax Microplate Reader (Molecular Devices, Sunnyvale, CA, EUA).

### Gene expression analysis by qPCR array

HCT116 cells were plated in bottles tissue culture (7x10^4^ cells/mL). After 12h of incubation with the complexes, total RNA was isolated from the cells using the RNeasy Plus mini kit (Qiagen, Hilden, Germany) according to the manufacturer’s instructions. The RNA was evaluated by fluorimetry (QuBit™, Life Technologies, Camarillo, CA, EUA). RNA reverse transcription was performed using Superscript VILO™ (Invitrogen Corporation, Waltham, MA, USA). A 96-well plate TaqMan® Array Human Molecular Mechanisms of Cancer (ID 4418806, Applied Biosystems™, Foster City, CA, EUA) was used for the gene expression study by qPCR. The reactions were conducted in the ABI ViiA7 (Applied Biosystems™, Foster City, CA, EUA). The cycle conditions comprised 2 min at 50 °C, 20 s at 95 °C, then 40 cycles of 3 s at 95 °C and 30 s at 60 °C. The relative quantification (RQ) of mRNA expression were calculated by 2^-ΔΔCT^ method [64] using the Gene Expression Suite™ Software (Applied Biosystems™, Foster City, CA, EUA) and the cells treated with the negative control (0.1% of a solution containing 70% sorbitol, 25% tween 80 and 5% water) was used as calibrator. The *GAPDH*, *18S* and *HPRT1* genes were used for normalization. All experiments were performed in DNase/RNase free conditions. The genes were considered to be upregulated if RQ ≥ 2, which means that the gene expression in the compound-treated cells was at least twice that in the negative control-treated cells. Similarly, the genes were considered to be downregulated if RQ ≤ 0.5, which means that the gene expression in the compound-treated cells was half or less than half that in the negative control-treated cells.

### Statistical analysis

Data are presented as mean ± S.E.M. or IC_50_ values and their 95% confidence intervals (CI 95%) obtained by nonlinear regression. Differences between experimental groups were compared using analysis of variance (ANOVA) followed by the Student–Newman–Keuls test (*p* < 0.05). All statistical analyses were performed using GraphPad (Intuitive Software for Science, San Diego, CA, USA).

## SUPPLEMENTARY MATERIALS FIGURES AND TABLES





## Abbreviations

3D: three-dimensional
ATCC: american type culture collection
CCND1: cyclin D1
CDKN2A: cyclin dependent kinase inhibitor 2A
ConA: concanavalin A
CV: cyclic voltammetry
ctDNA: calf thymus DNA
DAF-FM diacetate: 4-amino-5-methylamino-2’-,7’-difluofluorescein diacetate
DCF-DA: 2′,7′-dichlorofluorescin diacetate
DPV: differential pulse voltammetry
FADD: fas associated via death domain
FOS: fos proto-oncogene
FSC: forward light scatter
GSH: reduced glutathione
JUN: jun proto-oncogene, AP-1 transcription factor subunit
NAC: N-acetyl-L-cysteine
NFKBIA: NF-κB inhibitor alpha
PBMC: peripheral blood mononuclear cells
PF_6_^-^: hexafluorophosphate anion
ROS: reactive oxygen species
RQ: relative quantification
SCC: side scatter
SI: selectivity index
SHC1: SHC adaptor protein 1
TBAP: tetrabutylammonium perchlorate
TP53: tumor protein p53
Z-VAD(OMe)-FMK: Z-Val-Ala-Asp-(OMe)-fluoromethyl ketone
