# Supplementary material for: Novel piplartine-containing ruthenium complexes: synthesis, cell growth inhibition, apoptosis induction and ROS production on HCT116 cells
Source: Oncotarget. 2017 Nov 1;8(61):104367–92. doi: 10.18632/oncotarget.22248 (PMC5732813; doi:10.18632/oncotarget.22248)
Supplement: Supplementary file 1 [file oncotarget-08-104367-s001.pdf]

Novel piplartine-containing ruthenium complexes: synthesis, cell growth inhibition, apoptosis induction and ROS production on HCT116 cells

SUPPLEMENTARY MATERIALS

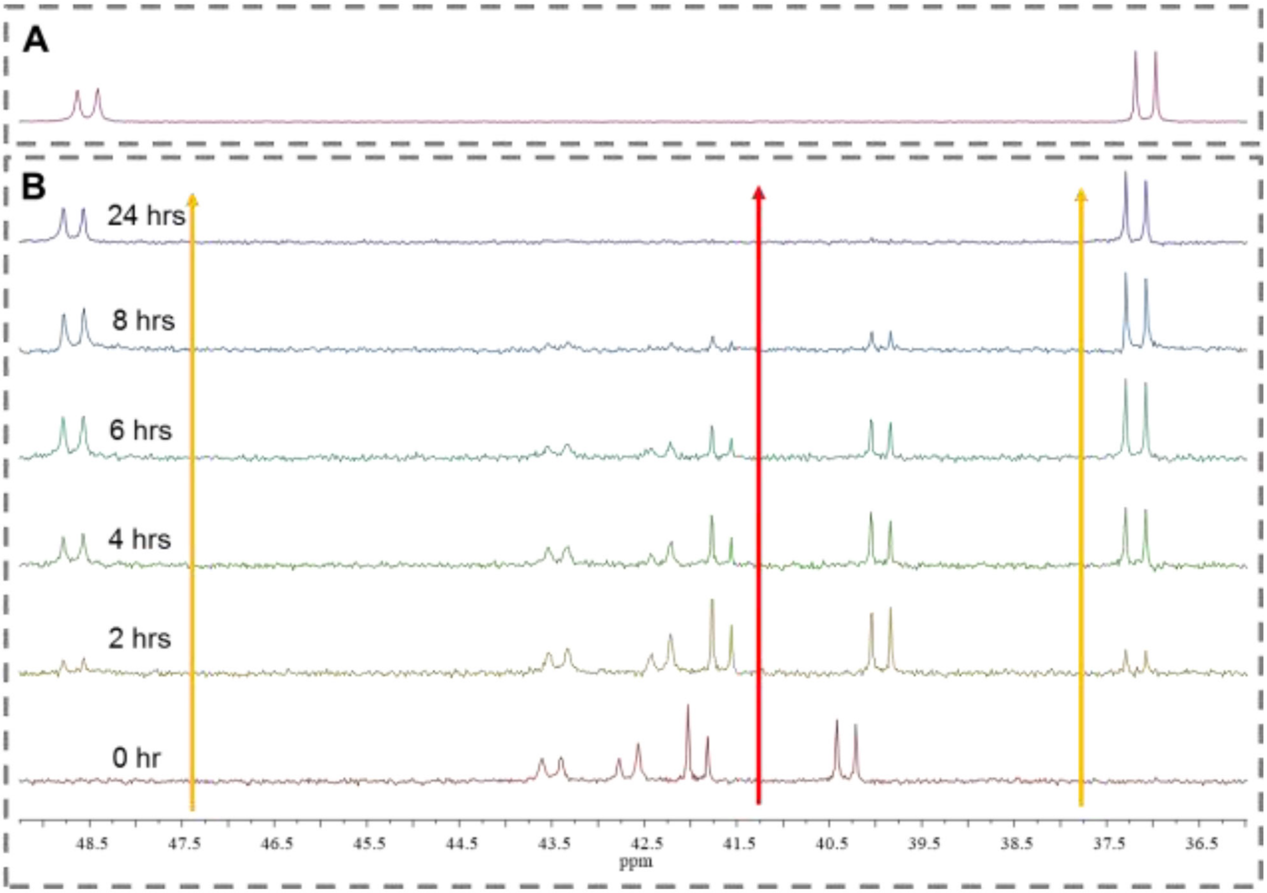

Supplementary Figure 1: (A) $^{31}P\{^{1}H\}$ NMR spectra of the complex $[RuCl_2(dppb)(bipy)]$ after the addition of $AgPF_6$, in the mixture acetone/water (1:9). **(B)** $^{31}P\{^{1}H\}$ NMR spectra (0 – 24 h) of complex **2** in the mixture acetone/water (1:9). The red arrow shows the consumption of complex **2** and the yellow arrow show the resulting product.

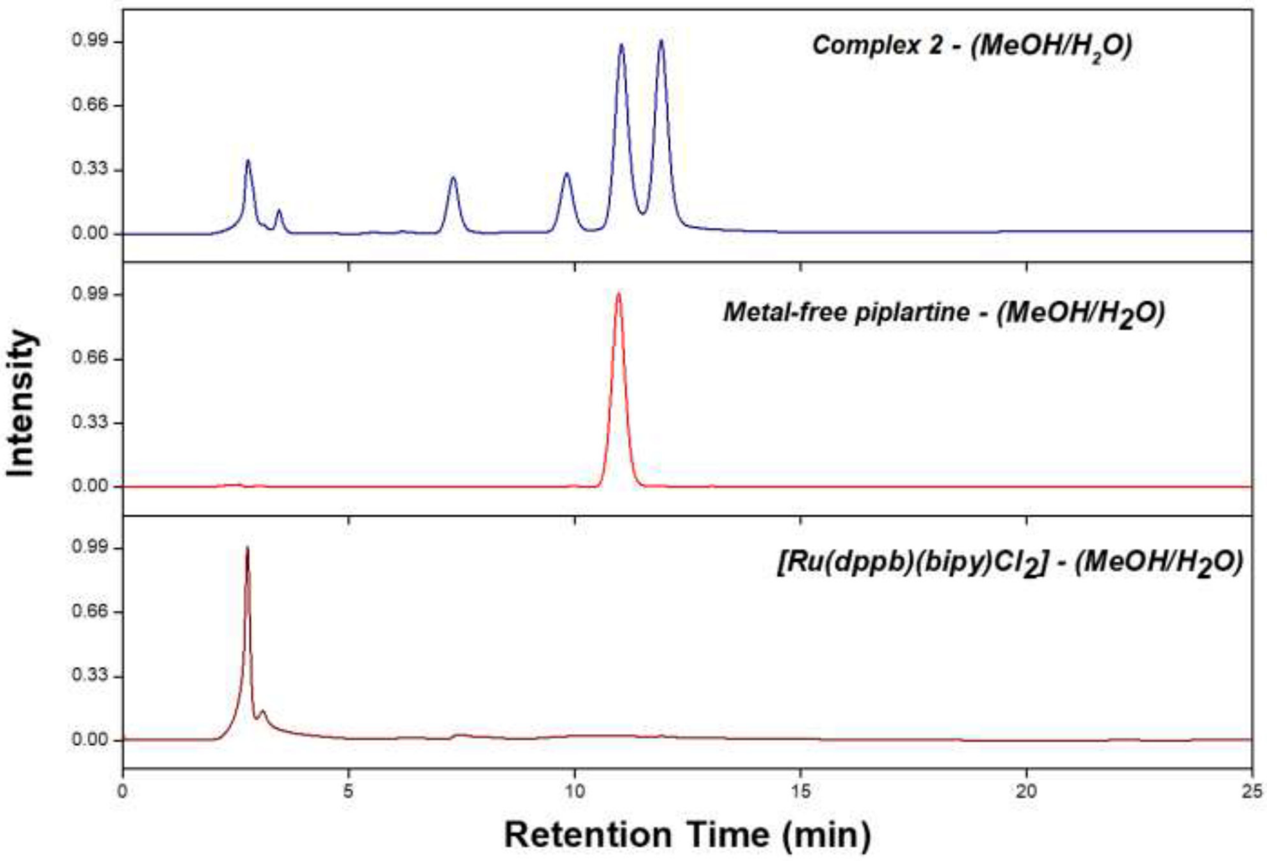

Supplementary Figure 2: HPLC chromatogram of complex 2, precursor [Ru(dppb)(bipy)Cl$_2$] and metal-free piplartine, using methanol (MeOH)/water (H_2O) (57/43) as mobile phase at isocratic model and the column ODS-C18 (5 mm; 250 x 4.6 mm; Shimadzu).

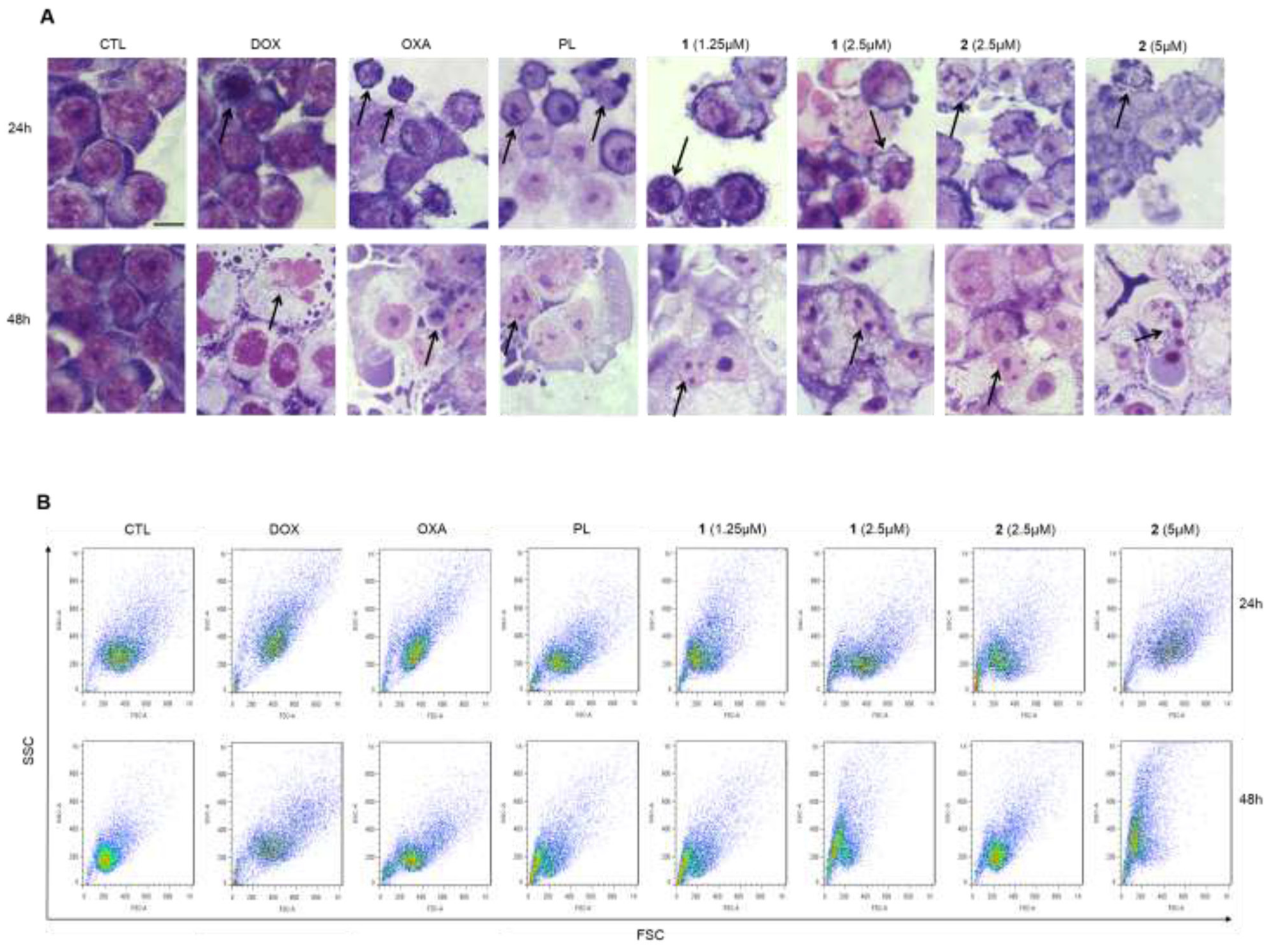

Supplementary Figure 3: Effect of piplartine-containing ruthenium complexes in the morphologic analysis of HCT116 cells after 24 and 48h of incubation. (A) Cells stained with may-grunwald-giemsa and examined by light microscopy (bar = 20 µm). Arrows indicated cells with fragmented DNA. **(B)** Light scattering features determined by flow cytometry. For flow cytometry analysis, 10,000 events were evaluated per experiment and cellular debris was omitted from the analysis. The negative control (CTL) was treated with the vehicle (0.1% of a solution containing 70% sorbitol, 25% tween 80 and 5% water) used for diluting the compounds tested. Doxorubicin (DOX, 1 µM), oxaliplatin (OXA, 3 µM) and piplartine (PL, 10 µM) were used as the positive controls. FSC: forward scatter; SCC: side scatter.

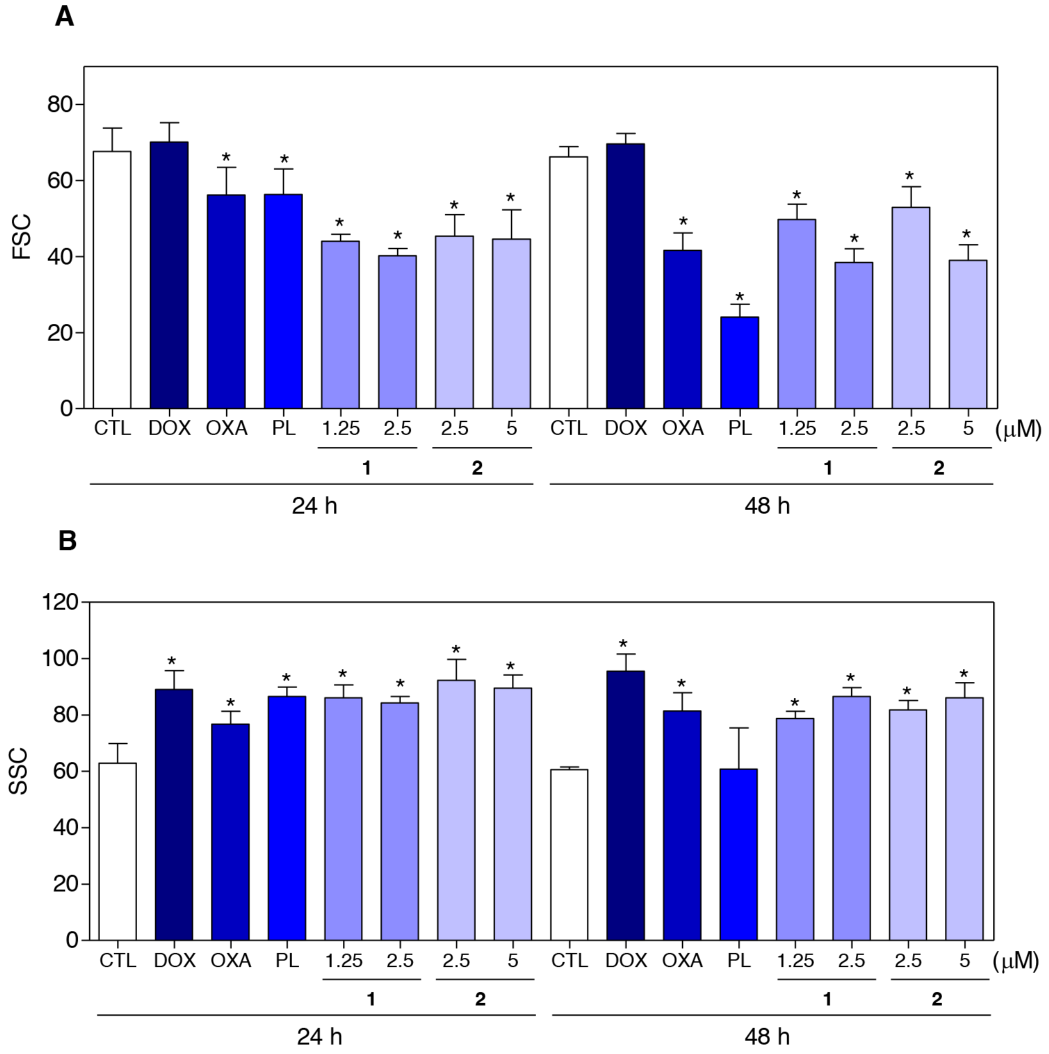

Supplementary Figure 4: Effect of piplartine-containing ruthenium complexes in the morphologic analysis of HCT116 cells after 24 and 48h of incubation. (A) Quantification of forward light scatter (FSC) determined by flow cytometry. **(B)** Quantification of side scatter (SCC) determined by flow cytometry. Ten thousands events were evaluated per experiment and cellular debris was omitted from the analysis. The negative control (CTL) was treated with the vehicle (0.1% of a solution containing 70% sorbitol, 25% tween 80 and 5% water) used for diluting the compounds tested. Doxorubicin (DOX, 1 µM), oxaliplatin (OXA, 3 µM) and piplartine (PL, 10 µM) were used as the positive controls.

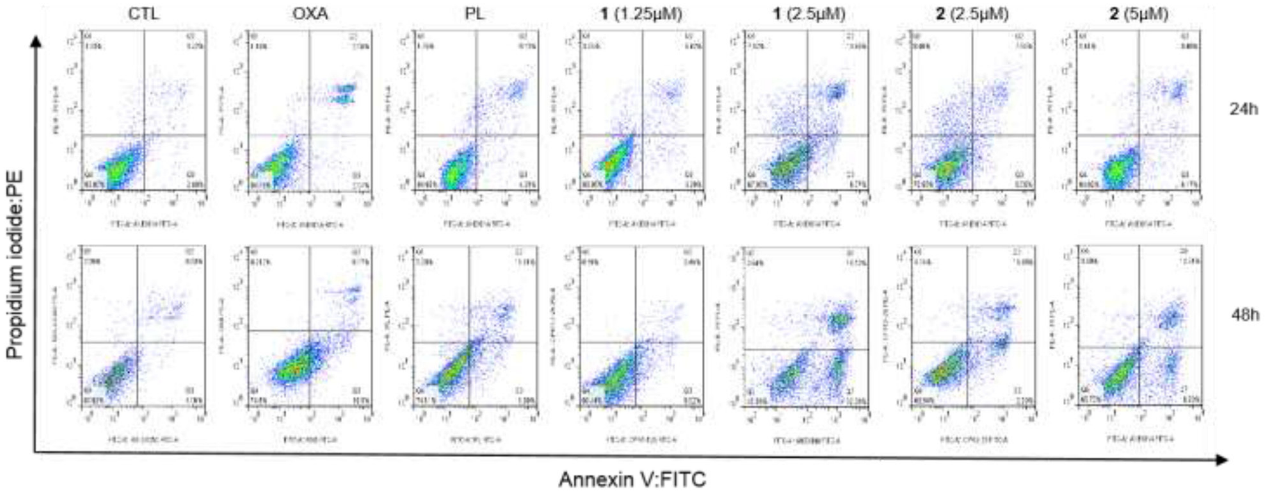

Supplementary Figure 5: Effect of piplartine-containing ruthenium complexes in the induction of apoptosis on HCT116 cells determined by flow cytometry using annexin V-FITC/PI staining after 24 and 48h of incubation. The data show the representative flow cytometry dot plots showing the percentage of cells in viable, early apoptotic, late apoptotic and necrotic stages. The negative control (CTL) was treated with the vehicle (0.1% of a solution containing 70% sorbitol, 25% tween 80 and 5% water) used for diluting the compounds tested. Oxaliplatin (OXA, 3 μM) and piplartine (PL, 10 μM) were used as the positive controls. Ten thousand events were evaluated per experiment and cellular debris was omitted from the analysis.

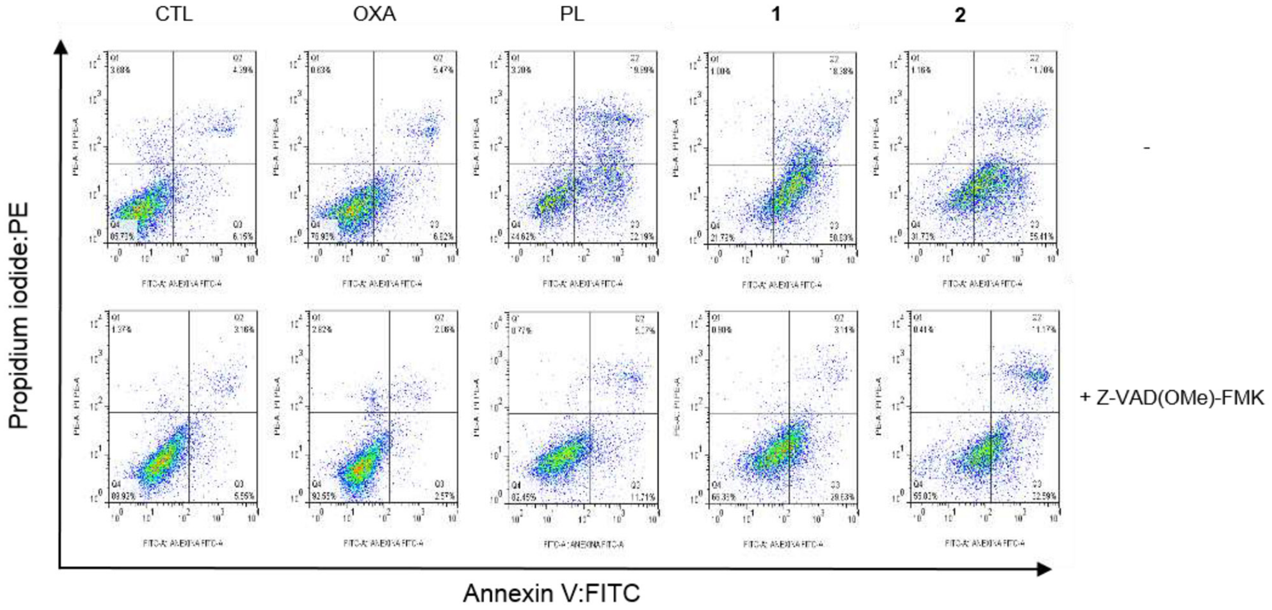

Supplementary Figure 6: Effect of the pan-caspase inhibitor (Z-VAD(OMe)-FMK) in the apoptosis induced by piplartine-containing ruthenium complexes on HCT116 cells determined by flow cytometry using annexin V-FITC/PI staining. The data show the representative flow cytometry dot plots showing the percentage of cells in viable, early apoptotic, late apoptotic and necrotic stages. The cells were pre-treated for 2 h with 50 μM Z-VAD(OMe)-FMK, then incubated with the complexes in the established concentration (2.5 μM for complex **1** and 5 μM for complex **2**) for 48 h. The negative control (CTL) was treated with the vehicle (0.1% of a solution containing 70% sorbitol, 25% tween 80 and 5% water) used for diluting the compounds tested. Oxaliplatin (OXA, 3 μM) and piplartine (PL, 10 μM) were used as the positive controls. Ten thousand events were evaluated per experiment and cellular debris was omitted from the analysis.

Supplementary Table 1: Selectivity index of piplartine-containing ruthenium complexes

Cancer cells	Non-cancer cells									
	MRC					PBMC				
	DOX	OXA	PL	1	2	DOX	OXA	PL	1	2
HCT116	6.5	0.3	2.7	2.0	1.1	27.0	2.3	5.3	1.1	0.6
HepG2	6.5	0.6	2.7	2.0	3.3	27.0	4.3	5.4	1.1	1.7
HSC-3	2.6	0.4	2.3	5.7	4.8	10.8	2.8	4.6	3.0	2.5
SCC-4	0.6	0.2	1.1	1.1	1.2	2.6	1.2	2.2	0.6	0.6
SCC-9	0.5	N.d	1.0	0.8	0.9	2.1	N.d	2.1	0.4	0.5
HL-60	6.5	2.2	1.3	1.0	1.4	27.0	15.7	2.6	0.5	0.7
K-562	1.3	1.3	0.9	1.0	1.1	5.4	9.4	1.8	0.5	0.6
B16-F10	65.0	0.6	1.6	1.2	1.5	270.0	4.3	3.2	0.6	0.8

Data are presented the selectivity index (SI) calculated using the following formula: SI = IC_{50}[non-cancer cells]/IC_{50}[cancer cells]. Cancer cells: HCT116 (human colon carcinoma); HepG2 (human hepatocellular carcinoma); HSC-3 (human oral squamous cell carcinoma); SCC-4 (human oral squamous cell carcinoma); SCC-9 (human oral squamous cell carcinoma); HL-60 (human promyelocytic leukemia); K-562 (human chronic myelogenous leukemia); and B16-F10 (murine melanoma). Non-cancer cells: MRC-5 (human lung fibroblast) and PBMC (human peripheral blood mononuclear cells). Doxorubicin (DOX), oxaliplatin (OXA) and piplartine (PL) were used as the positive controls. N.d. Not determined.

Supplementary Table 2: The effect of piplartine-containing ruthenium complexes on gene expression of HCT116 cells.

See Supplementary File 1
