# Supplementary material for: Novel piplartine-containing ruthenium complexes: synthesis, cell growth inhibition, apoptosis induction and ROS production on HCT116 cells
Source: Oncotarget. 2017 Nov 1;8(61):104367–92. doi: 10.18632/oncotarget.22248 (PMC5732813; doi:10.18632/oncotarget.22248)
Supplement: Supplementary file 2 [file oncotarget-08-104367-s002.doc]

**Supplementary Table 2: The effect of piplartine-containing ruthenium complexes on gene expression of HCT116 cells**

| Gene  symbol | RQ | | | |
| --- | --- | --- | --- | --- |
|  | DOX | PL | **1** | **2** |
| *ABL1* | 0.912 | 1.144 | 1.302 | 0.863 |
| *AKT1* | 1.840 | 1.285 | 1.469 | 0.642 |
| *AKT2* | 3.877 | 1.068 | 1.663 | 0.592 |
| *APC* | 0.908 | 1.236 | 0.972 | 0.576 |
| *BAX* | 3.274 | 2.160 | 1.572 | 0.739 |
| *BCAR1* | 1.809 | 0.705 | 1.687 | 0.596 |
| *BCL2* | 3.090 | 1.049 | 2.367 | 0.748 |
| *BCL2L1* | 2.391 | 0.822 | 1.936 | 0.739 |
| *BCL2L11* | 1.152 | 1.536 | 1.710 | 0.904 |
| *BID* | 1.757 | 0.528 | 0.743 | 0.671 |
| *BRAF* | 0.513 | 1.512 | 1.072 | 0.781 |
| *CASP8* | 1.043 | 0.915 | 1.583 | 0.930 |
| *CASP9* | 0.530 | 1.065 | N.d. | 0.828 |
| *CCND1* | 1.124 | 0.669 | 1.153 | 0.461 |
| *CCND2* | N.d. | N.d. | N.d. | N.d. |
| *CCND3* | 1.438 | 1.144 | 1.447 | 0.723 |
| *CCNE1* | 0.894 | 1.418 | 1.075 | 0.893 |
| *CDC42* | 1.271 | 2.542 | 1.755 | 1.411 |
| *CDH1* | 7.107 | 2.494 | 3.659 | 1.098 |
| *CDK2* | 1.257 | 0.722 | 1.467 | 0.693 |
| *CDK4* | 0.983 | 0.873 | 1.390 | 0.898 |
| *CDKN1A* | 2.853 | 2.847 | 1.331 | 0.996 |
| *CDKN1B* | 0.766 | 1.545 | 1.039 | 0.783 |
| *CDKN2A* | 1.793 | 1.575 | 2.469 | 1.153 |
| *CDKN2B* | N.d. | N.d. | N.d. | N.d. |
| *COL1A1* | N.d. | N.d. | N.d. | N.d. |
| *CRK* | 0.867 | 0.381 | 1.184 | 0.758 |
| *CTNNB1* | 0.939 | 1.026 | 1.131 | 0.877 |
| *CYCS* | 0.993 | 0.954 | 1.961 | 1.043 |
| *DVL1* | 1.252 | 0.913 | 1.576 | N.d. |
| *E2F1* | 1.018 | 0.764 | 1.631 | 0.736 |
| *EGFR* | 0.831 | 1.173 | 1.294 | 11,276.298 |
| *ELK1* | 1.263 | 0.625 | 1.275 | 0.628 |
| *ERBB2* | 2.605 | 1.273 | 1.941 | 1.006 |
| *FADD* | 1.049 | 0.369 | 0.556 | 74.142 |
| *FAS* | 2.372 | 1.371 | 1.496 | 1.037 |
| *FASLG* | N.d. | N.d. | N.d. | N.d. |
| *FGF2* | 0.625 | 0.854 | 1.120 | 1.003 |
| *FN1* | 3.575 | 1.952 | 3.841 | N.d. |
| *FOS* | 11.834 | 30.985 | 11.943 | N.d. |
| *FYN* | 1.248 | 1.675 | 2.474 | 1.169 |
| *FZD1* | 0.994 | 0.953 | 2.253 | 0.860 |
| *GRB2* | 0.761 | 1.219 | 0.961 | 0.693 |
| *GSK3B* | 1.342 | 1.249 | 1.191 | 0.738 |
| *HGF* | N.d. | N.d. | N.d. | N.d. |
| *HRAS* | 0.691 | 0.928 | 0.777 | 0.561 |
| *IGF1* | N.d. | N.d. | N.d. | N.d. |
| *IGF1R* | 1.389 | 2.082 | 2.283 | 0.677 |
| *ITGA2B* | 4.514 | 2.963 | 3.092 | 0.135 |
| *ITGAV* | 1.265 | 0.993 | 1.426 | 1,149.015 |
| *ITGB1* | 0.489 | 0.784 | 0.838 | 0.579 |
| *ITGB3* | 5.579 | N.d. | N.d. | 1.243 |
| *JUN* | 4.073 | 3.837 | 3.708 | 2.306 |
| *KDR* | N.d. | 8.270 | 4.404 | 1.408 |
| *KIT* | N.d. | N.d. | N.d. | N.d. |
| *KRAS* | 0.951 | 0.133 | 0.886 | 0.979 |
| *LEF1* | N.d. | N.d. | N.d. | N.d. |
| *MAP2K1* | 0.681 | 0.759 | 0.822 | 2,107.239 |
| *MAP3K5* | 1.006 | 0.609 | 0.308 | 4.314 |
| *MAPK1* | 0.903 | 1.098 | 1.305 | 1.062 |
| *MAPK14* | 0.786 | 1.009 | 1.008 | 1.145 |
| *MAPK3* | 2.758 | 1.726 | 2.014 | 1.853 |
| *MAPK8* | 1.185 | 0.887 | 1.411 | 0.623 |
| *MAX* | N.d. | 3.051 | 3.614 | 0.912 |
| *MDM2* | 1.479 | 0.901 | 0.794 | 1.272 |
| *MYC* | 0.876 | 0.271 | 1.065 | 0.796 |
| *NFKB1* | 0.784 | 0.870 | 1.288 | 0.699 |
| *NFKB2* | 1.250 | 0.818 | 1.340 | 1.536 |
| *NFKBIA* | 2.326 | 1.989 | 2.007 | 0.894 |
| *NRAS* | 0.556 | 0.600 | 1.178 | 0.923 |
| *PIK3CA* | 0.409 | 0.910 | 0.880 | 0.565 |
| *PIK3R1* | 1.999 | 2.352 | 2.456 | 1.299 |
| *PTEN* | 1.750 | 0.501 | 2.037 | 0.776 |
| *PTK2* | 1.182 | 0.616 | 1.561 | 2.357 |
| *PTK2B* | 3.368 | 2.265 | 2.270 | 1.024 |
| *RAC1* | 0.845 | 0.731 | 1.059 | 2.676 |
| *RAF1* | 2.027 | 0.574 | 1.951 | 0.909 |
| *RB1* | 0.532 | 1.314 | 1.003 | 0.821 |
| *RELA* | 0.862 | 1.240 | 1.390 | 0.845 |
| *RHOA* | 0.900 | 0.979 | 1.129 | 1.017 |
| *SHC1* | 0.640 | 0.243 | 0.436 | N.d. |
| *SMAD4* | 0.963 | 0.942 | 1.409 | 2.050 |
| *SOS1* | 0.741 | 0.851 | 1.161 | 0.188 |
| *SPP1* | 1.165 | 1.338 | 2.500 | 10.094 |
| *SRC* | 1.383 | 1.076 | 1.584 | 0.801 |
| *TCF3* | 1.135 | 0.774 | N.d. | 0.608 |
| *TGFB1* | 0.495 | 0.947 | 0.911 | 0.868 |
| *TGFBR1* | 1.216 | 0.545 | 1.324 | 0.781 |
| *TGFBR2* | 0.914 | 1.177 | 2.358 | 1.085 |
| *TP53* | 3.250 | 1.182 | 3.478 | 1.466 |
| *VEGFA* | 0.610 | 1.245 | 1.664 | 1.321 |
| *WNT1* | N.d. | N.d. | N.d. | N.d. |

HCT116 cells were treated with 2.5 µM of complex **1** and 5 µM of complex **2** for 12 h. The negative control was treated with the vehicle (0.1% of a solution containing 70% sorbitol, 25% tween 80 and 5% water) used for diluting the compounds tested. Doxorubicin (DOX, 1µM) and piplartine (PL, 10µM) were used as the positive controls. After treatment, total RNA was isolated and reverse transcribed. Gene expression was detected using the 96-well plate TaqMan® Array Human Molecular Mechanisms of Cancer. *GAPDH*, *18S* and *HPRT1* genes were used as endogenous gene for normalization. Values represent the relative quantitation (RQ) compared with the calibrator (cells treated with the negative control, RQ = 1.0). The genes were considered to be upregulated if RQ ≥ 2 and were considered to be downregulated if RQ ≤ 0.5. N.d. Not determinated.
